# The DNA repair pathway as a therapeutic target to synergize with trastuzumab deruxtecan in HER2-targeted antibody–drug conjugate–resistant HER2-overexpressing breast cancer

**DOI:** 10.1186/s13046-024-03143-3

**Published:** 2024-08-21

**Authors:** Jangsoon Lee, Kumiko Kida, Jiwon Koh, Huey Liu, Ganiraju C. Manyam, Young Jin Gi, Dileep R. Rampa, Asha S. Multani, Jing Wang, Gitanjali Jayachandran, Dae-Won Lee, James M. Reuben, Aysegul Sahin, Lei Huo, Debu Tripathy, Seock-Ah Im, Naoto T. Ueno

**Affiliations:** 1 Section of Translational Breast Cancer Research and Department of Breast Medical Oncology, The University of Texas MD Anderson Cancer Center, Houston, TX USA; 2 Cancer Research Institute, Seoul National University, Seoul, Republic of Korea; 3 Department of Pathology, Seoul National University Hospital, Seoul National University College of Medicine, Seoul, Republic of Korea; 4 Department of Bioinformatics and Computational Biology, The University of Texas MD Anderson Cancer Center, Houston, TX USA; 5 Department of Genetics, The University of Texas MD Anderson Cancer Center, Houston, TX USA; 6 Department of Hematopathology, The University of Texas MD Anderson Cancer Center, Houston, TX USA; 7 Department of Pathology, The University of Texas MD Anderson Cancer Center, Houston, TX USA; 8 Department of Internal Medicine, Seoul National University Hospital, Seoul National University College of Medicine, 101 Daehak-Ro Jongro-Gu, Seoul, 03080 Republic of Korea; 9 Cancer Biology and Therapeutics, University of Hawai‘I Cancer Center, 701 Ilalo Street, Room 622, Honolulu, HI 96813 USA; 10 Present address: Cancer Biology Program, University of Hawai’I Cancer Center, 701 Ilalo Street, Honolulu, HI 96813 USA; 11 Present Address: Present address: Department of Breast Surgical Oncology, St. Luke’s International Hospital, 9-1, Akashicho, Chuouku, Tokyo, 104-8560 Japan

**Keywords:** HER2+ breast cancer, HER2 antibody–drug conjugates, DNA damage repair pathway, T-DXd, HER2-directed ADC resistance

## Abstract

**Background:**

Anti-HER2 therapies, including the HER2 antibody–drug conjugates (ADCs) trastuzumab emtansine (T-DM1) and trastuzumab deruxtecan (T-DXd), have led to improved survival outcomes in patients with HER2-overexpressing (HER2+) metastatic breast cancer. However, intrinsic or acquired resistance to anti-HER2–based therapies remains a clinical challenge in these patients, as there is no standard of care following disease progression. The purpose of this study was to elucidate the mechanisms of resistance to T-DM1 and T-DXd in HER2+ BC patients and preclinical models and identify targets whose inhibition enhances the antitumor activity of T-DXd in HER2-directed ADC-resistant HER2+ breast cancer in vitro and in vivo.

**Methods:**

Targeted DNA and whole transcriptome sequencing were performed in breast cancer patient tissue samples to investigate genetic aberrations that arose after anti-HER2 therapy. We generated T-DM1 and T-DXd–resistant HER2+ breast cancer cell lines. To elucidate their resistance mechanisms and to identify potential synergistic kinase targets for enhancing the efficacy of T-DXd, we used fluorescence in situ hybridization, droplet digital PCR, Western blotting, whole-genome sequencing, cDNA microarray, and synthetic lethal kinome RNA interference screening. In addition, cell viability, colony formation, and xenograft assays were used to determine the synergistic antitumor effect of T-DXd combinations.

**Results:**

We found reduced HER2 expression in patients and amplified DNA repair–related genes in patients after anti-HER2 therapy. Reduced *ERBB2* gene amplification in HER2-directed ADC–resistant HER2+ breast cancer cell lines was through DNA damage and epigenetic mechanisms. In HER2-directed ADC–resistant HER2+ breast cancer cell lines, our non-biased RNA interference screening identified the DNA repair pathway as a potential target within the canonical pathways to enhance the efficacy of T-DXd. We validated that the combination of T-DXd with ataxia telangiectasia and Rad3-related inhibitor, elimusertib, led to significant breast cancer cell death in vitro (*P* < 0.01) and in vivo (*P* < 0.01) compared to single agents.

**Conclusions:**

The DNA repair pathways contribute to HER2-directed ADC resistance. Our data justify exploring the combination treatment of T-DXd with DNA repair–targeting drugs to treat HER2-directed ADC–resistant HER2+ breast cancer in clinical trials.

**Supplementary Information:**

The online version contains supplementary material available at 10.1186/s13046-024-03143-3.

## Introduction

Breast cancer (BC) is a heterogeneous disease that is classified into three subtypes that guide treatment: hormone receptor-positive, human epidermal growth factor receptor 2 positive (HER2+), and triple-negative. HER2, a member of the ErbB receptor tyrosine kinase family and an oncogene, is amplified or overexpressed in 15%-30% of BC cases [1] and activates oncogenic signaling pathways such as proliferation, angiogenesis, and metastasis by homodimerization or heterodimerization with other ErbB receptors, including EGFR, HER3, and HER4 [2]. HER2 overexpression is associated with higher rates of disease recurrence, brain metastasis, and mortality [3].

The HER2 oncogene is a well-defined BC biomarker for targeted therapies [4]. The humanized monoclonal antibody trastuzumab was the first targeted therapy for the HER2 protein, targeting its extracellular domain; as an advanced and adjuvant treatment with chemotherapy for patients with HER2+ BC, trastuzumab has been associated with a significant survival benefit [5]. Subsequent preclinical studies have led to other humanized monoclonal antibodies (e.g., pertuzumab, margetuximab) and small-molecule kinase inhibitors (e.g., lapatinib, neratinib, and tucatinib) targeting the intracellular kinase domain of HER2. Since anti-HER2 therapy enhances the therapeutic efficacy of chemotherapy, the antibody–drug conjugate (ADC) class of drugs was developed to maximize the cytotoxic effect of anti-HER2 therapy and chemotherapy via endocytosis selectively in HER2+ tumor cells [6]. Two such ADCs, ado-trastuzumab emtansine (T-DM1) and trastuzumab deruxtecan (T-DXd, also called DS8201a), were approved by the U.S. Food and Drug Administration (FDA) to treat HER2+ metastatic BC and unresectable or metastatic HER2-low BC. T-DXd is composed of an anti-HER2 antibody with the same amino acid sequence as trastuzumab, a cleavable tetrapeptide linker, and a membrane-permeable topoisomerase I inhibitor that is an exatecan (DX-8951f) derivative [7]. T-DXd showed enhanced tumor cell killing in a T-DM1–resistant HER2+ xenograft model and a low-HER2-expressing BC model [8]. In clinical trials of patients with HER2+ metastatic BC, T-DXd showed durable antitumor activity and progression-free survival in a T-DM1–pretreated population (DESTINY-Breast01 and DESTINY-Breast02 trials) [9, 10], and had better therapeutic efficacy than T-DM1 (DESTINY-Breast03 trial) [11]. T-DXd also resulted in significantly longer progression-free and overall survival duration than untargeted chemotherapy in patients with HER2-low metastatic BC (DESTINY-Breast04 trial) [12].

Despite the clinical benefits of HER2-directed ADC, BC often develops resistance to treatment. Understanding the mechanisms of innate or acquired resistance to HER2-directed ADC therapy and identifying potential novel therapeutic targets to overcome this resistance is important to improve outcomes in patients with metastatic HER2+ BC. In this study, we investigated mechanisms of resistance to T-DM1 and T-DXd in HER2+ BC patients and preclinical models and identified potential targets whose inhibition enhances the antitumor activity of T-DXd in HER2-directed ADC-resistant HER2+ BC in vitro and in vivo.

## Materials & methods

Detailed information regarding the sulforhodamine B cell proliferation assay, high-throughput RNA interference (RNAi) screening, the whole-genome sequencing analysis, karyotyping by G-banding and the genomic instability analysis, the fluorescence in situ hybridization analysis, the microarray analysis, the droplet digital PCR assay, and Western blotting is included in the electronic Supplementary Material.

### Patient data analysis after anti-HER2 and T-DM1 treatment

Ten patients with HER2+ metastatic BC that had progressed during treatment with T-DM1 and/or dual treatment with pertuzumab and trastuzumab were prospectively enrolled at MD Anderson (institutional review board [IRB] No: PA15-0499). All patients underwent biopsies from a primary or metastatic site before treatment with T-DM1 and/or pertuzumab and trastuzumab and after the development of clinical resistance to treatment. HER2 expression levels before and after therapy were compared using immunohistochemistry staining (IHC) and/or fluorescence in situ hybridization. Targeted next-generation sequencing was performed using the FoundationOne CDx assay (Foundation Medicine, Inc.) to identify gene alterations. Search Tool for the Retrieval of Interacting Genes/Proteins (STRING) software (V11, https://string-db.org/) was used for the functional enrichment analysis to find canonical pathways using gene alteration information. Patient characteristics are shown in Sup. Table 1 and details of clinicopathological features and treatments of patients are shown in Sup. Table 2.

### Targeted DNA sequencing on human BC samples before and after T-DXd treatment

Tissue samples from 16 patients at Seoul National University Hospital (SNUH), including pre- and post-T-DXd treatment metastatic BC samples from three patients, were used in a separate genomic analysis (SNUH IRB No. 2310–165-1480). Targeted DNA sequencing was performed using formalin-fixed paraffin-embedded (FFPE) samples, and the results were aligned to the reference genome (GRCh37/hg19) using Burrows-Wheeler Aligner (version 0.7.17) [13], followed by processing through the Genome Analysis Tool Kit (version 4.0.2.1) [14]. Clinicopathological features of patients treated with T-DXd shown in Sup. Table 3. A list of genes selected for targeted DNA sequencing is summarized in Sup. Table 4. We developed an in-house pipeline to call SNVs/indels by modifying SNVer (version 0.5.3) [15] and LoFreq (version 2.1.2) [16]. DELLY and Manta were used to detect translocations [17, 18]. For copy number variation (CNV) estimation, a panel of normal samples was established by sequencing non-neoplastic samples. CNV calls were made through an in-house developed process based on CNVKit [19]; amplifications were called when copy number estimates reached ≥ six copies, and homozygous deletions were called at zero copies. ANNOVAR and SnpEff (version 4.3) were used for annotation [20, 21]. All CNV and structural variations were manually reviewed with the Integrative Genomics Viewer (IGV) [22]. To filter out possible germline variants, those with an allele frequency of more than 0.1% in the Genome Aggregation Consortium (gnomAD) East Asian database [23], Korean Reference Genome Database [24], and Korean Variant Archive were discarded [25].

### RNA-seq and gene set enrichment analysis

RNA-seq was performed on six paired samples from SNUH, which included three samples obtained before the T-DXd treatment and three obtained at the time of disease progression after T-DXd. Sequencing libraries were prepared using SureSelect RNA Direct_Human (Agilent Technologies) and sequenced on the Illumina NovaSeq 6000 (Macrogen, Seoul, Republic of Korea) using the paired-end 2 × 100-bp option. After quality assessment of raw FASTQ files using FastQC (v.0.11.7), adapter sequences in sequencing reads were trimmed using Trimmomatic (v.0.38) [26]. Reads were aligned using HISAT2 (v.2.1.0) [27] and assembled and quantified using StringTie (v.2.1.3b) [28] as read counts.

The raw read counts were normalized using DESeq2 [29], and the normalized read counts were used as inputs in the gene set enrichment analysis (GSEA) [30]. GSEA was performed between pre- and post-TDX-d samples, and the analysis was performed by gene set permutation mode due to the small sample size [31]. Gene sets from the Molecular Signatures Database (MSigDB, http://software.broadinstitute.org/gsea/msigdb) [32] were used. A cut-off false discovery rate (FDR) q-value of ≤ 0.25 was used to define significant enrichment.

### Cell lines and reagents

HCC1954, SKBR3, BT474, HCC1419, and MDA-MB-231 BC cells were purchased from the American Type Culture Collection (Manassas, VA, USA). SUM190 BC cells were purchased from Asterand Bioscience (Detroit, MI, USA). KPL4 BC cells were provided by Kawasaki Medical School (Okayama, Japan). HCC1954, SKBR3, BT474, and HCC1419 cells were maintained in Roswell Park Memorial Institute 1640 medium (Sigma-Aldrich, St. Louis, MO, USA). MDA-MB-231 cells were maintained in Dulbecco’s modified Eagle’s medium/F-12 medium (Sigma-Aldrich). SUM190 and KPL4 cells were maintained in Ham’s F-12 medium (Sigma-Aldrich) supplemented with 5 µg/mL of insulin (Thermo Fisher Scientific Inc., Waltham, MA, USA), and 1 µg/mL of hydrocortisone (Sigma-Aldrich). All media were supplemented with 10% fetal bovine serum (GenDEPOT, Katy, TX, USA) and 1% antibiotic/antimycotic (Sigma-Aldrich). All cell lines were validated by DNA typing at the MD Anderson Characterized Cell Line Core and confirmed to be free of *Mycoplasma* using the MycoAlert Mycoplasma Detection Kit (Lonza, Morristown, NJ, USA).

T-DM1-resistant (TDM1R) or T-DXd–resistant (TDXdR) HER2+ BC cell lines were established by a continuous treatment/recovery cycle with HER2-directed ADC. In brief, 1 × 10^5^ cells were seeded into a 100-mm cell culture dish and incubated overnight. The next day, the cells were treated with T-DM1 or T-DXd at the IC_80_ concentration, as determined by a sulforhodamine B cell proliferation assay. After 3–5 days of incubation, the culture media was replaced with fresh media without HER2-directed ADC until cells recovered about 30%-50% cell confluency. The treatment/recovery cycle was repeated with a 10%-25% increased HER2-directed ADC concentration until BC cells were resistant to at least 2 µg/mL of HER2-directed ADC.

Ataxia telangiectasia and Rad3-related inhibitor elimusertib (also known as BAY 1895344), abemaciclib, AZD1775, BYL719, olaparib, and TAS-119 were purchased from MedChemExpress (Monmouth Junction, NJ, USA). T-DM1 was purchased from the MD Anderson Pharmacy division. T-DXd and DXd were provided by Daiichi Sankyo Co., Ltd. (Tokyo, Japan).

### Xenograft studies

For the tumorigenicity studies, 315 of 4- to 6-week-old female athymic nude mice (#002019, Jackson Laboratory, Bar Harbor, ME, USA) were housed under pathogen-free conditions and treated per National Institutes of Health guidelines. To establish TDM1R and TDXdR BC cell line xenografts, cell suspensions (4 × 10^6^ cells/100 µL) in 50% Matrigel were injected into one site in each mouse's abdominal mammary fat pad. When tumors were approximately 100–250 mm^3^, mice were randomly distributed into four groups (*n* = 10–15 mice per group) to achieve similar average tumor volumes (200–250 mm^3^) and standard deviation across the groups. T-DXd (10 mg/kg) was administered one time on Day 0 via intravenous injection as previously described [7, 8], and elimusertib was administered twice a day in a cycle of 3 days on and 4 days off via oral gavage in 40% propylene glycol 400 (Lab Alley, Austin, TX, USA) plus 10% ethanol. Tumor volume [*V* = 0.5 × (*L* × *W*^2^)] was measured by caliper and body weight was measured twice weekly. Following euthanasia using carbon dioxide inhalation, tumor samples were collected, and animal remains were handled in accordance with institutional biohazard waste disposal protocols.

### IHC staining

Xenograft tumor tissues were fixed in 10% neutral-buffered formalin and embedded in paraffin. The Sects. (5-μm thick) were deparaffinized in xylene for 2 min three times, rehydrated in graded alcohols for 1 min, and washed in distilled water. IHC staining was performed using VECTASTAIN® Elite® ABC-HRP Kit (PK-7200, Newark, CA, USA) for manual staining or the Leica BOND-RX^m^ automated IHC staining system (Leica Biosystems, Wetzlar, Germany) through the MD Anderson Department of Veterinary Medicine and Surgery Histology Core. The Leica Refine polymer kit (#DS-9800) was used for detection in IHC. The slides were then incubated with the following antibodies: anti–Ki-67 (#RM-9106, 1:100, Thermo Fisher Scientific), anti-HER2 (#APA342AA, Biocare Medical, Pacheco, CA, USA), anti–phospho-ATR (#2933, 1:200, Cell Signaling Technology, Danvers, MA, USA), or anti–cleaved poly(ADP-ribose) polymerase (PARP, #5625, 1:100, Cell Signaling Technology). Immunostained slides were scanned using an Aperio AT2 slide scanner (Leica Biosystems) and captured at 20 × magnification using Aperio ImageScope software (Leica Biosystems). HER2 expression was measured on both membrane and cytosol. pH2AX, pATR, or Ki-67 was evaluated on nucleus expression only.

### Statistical analysis

The cell proliferation rate was summarized with descriptive statistics (mean, median, and quartiles) and box plots for each treatment group. A two-tailed unpaired Student’s *t*-test was used for statistical analysis using GraphPad Prism (version 9, GraphPad Software, Boston, MA, USA). For the evaluation of xenograft assays, treatment groups were compared at the indicated time points using multiple *t*-test analysis of multiple comparisons testing using GraphPad Prism. *P* ≤ 0.05 was considered significant.

## Results

### *ERBB2* gene reduction and alteration of DNA repair were observed after receipt of HER2-targeted therapies in patients with HER2+ BC

**Fig. 1:**
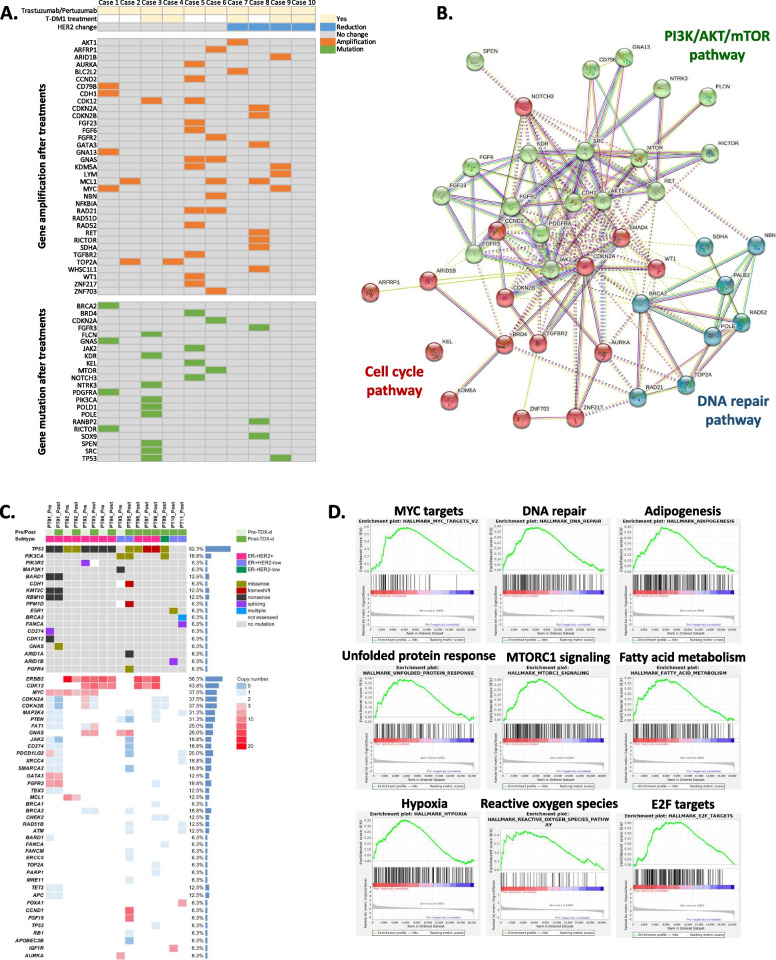
DNA repair pathways are activated after HER2-targetd drug treatment in patients with HER2+ BC. **A** Targeted WGS was performed on 10 paired patient samples after treatment with trastuzumab/pertuzumab or T-DM1. The table shows gene amplification or variation after treatment. **B** The gene list was analyzed using STRING software (version 11.0) to show similar categories by functions of genes. In the context of the STRING analysis, k-means clustering was applied to identify groups of genes with similar behavior. Each color indicates co-regulated gene modules related to specific canonical signaling pathways. **C** Targeted DNA sequencing was performed to identify gene alteration profiling from five pairs before and after T-DXd treatment and six after T-DXd treatment in BC patients tissue samples. Genomic DNA was collected from FFPE tissue samples. **D** Gene expression analysis in three pairs before and after T-DXd treatment in HER2+ BC patients tissue samples. Total RNA was collected from FFPE tissue slides, and an RNA-seq analysis was conducted

We analyzed paired pre- and post-treatment samples from 10 patients who underwent anti-HER2 treatment and chemotherapy for metastatic BC and experienced disease progression during treatment. Of these, 5 patients received T-DM1. HER2 expression was reduced in the post-treatment biopsy in 4 of the 10 patients, including 3 of the 5 patients who received T-DM1 therapy after trastuzumab and pertuzumab combination therapy (Fig. 1A). The reduction in HER2 expression in these patients was accompanied by the loss of *ERBB2* amplification. At disease progression after T-DM1, 3 of 5 patients (60%) had lost *ERBB2* amplification. In contrast, in patients treated with trastuzumab and pertuzumab alone, only 1 of the 5 (20%) had lost HER2 amplification.

In patients with reduced HER2 expression, heterogeneity across specimen sites was observed. In three cases, HER2 expression was negative in some specimens, even in the pre-treatment biopsy—for example, HER2 was expressed in the primary BC tumor but not in the lymph nodes. In such cases, HER2 status was more likely to change after treatment. In contrast, in six cases, regardless of multiple biopsies, HER2 was consistently positive.

To explore the mechanism of HER2 loss and the development of resistance to anti-HER2 treatment, we analyzed genomic changes between the paired pre- and post-treatment samples, using the STRING database to identify protein–protein interactions. A pathway analysis showed multiple gene alterations after anti-HER2 treatment (Fig. 1B). In particular, genes related to the DNA repair pathway were amplified, including TOP2A, RAD21, RAD52, and MCL1. Of note, TOP2A, RAD21, and MCL1 were simultaneously upregulated in several cases. MCL1 amplification was observed in the three cases, including two cases with unchanged HER2 expression and one case with reduced expression. TOP2A amplification was observed in two cases with unchanged HER2 expression.

### Genetic alterations and gene expression profiles revealed upregulation of DNA repair pathway in patients with HER2+ BC after receipt of T-DXd

The most common genetic alterations in human BC samples from SNUH were *TP53* mutations (82.3%), *ERBB2* amplifications (56.3%), *CDK12* amplifications (43.8%), *MYC* amplifications (37.5%), and *CDKN2A/2B* copy number loss (37.5%) (Fig. 1C, Sup. Table 5, and Sup. Table 6). Some interesting features were noted when comparing the paired samples from the same patients (Sup. Table 5). While PT02, PT03, and PT04 had similar mutational profiles regardless of treatment history, we observed differences between pre- and post-T-DXd samples in PT01 and PT05. PT01 lost the *CD274* splicing mutation and *CDK12* truncation mutation during treatment. In contrast, PT01 gained the *GNAS* R201H hotspot activating mutation. PT05 harbored a *MAP3K1* nonsense mutation and *AURKA* amplification, which were not detectable after T-DXd treatment. However, the *TP53* R273C mutation emerged after T-DXd treatment, along with pathogenic mutations in *ARID1A* and *FGFR4*. In addition, copy number loss of *JAK2/CD274/PDCD1LG2* was noted in PT05_Post as well as amplifications of *CCND1/FGF19*.

Copy number losses of many DNA repair–related genes, including *BRCA2*, *RAD51B*, *ATM*, and *MRE11,* were also observed in PT05_Post, although their exact roles would require additional study. Interestingly, when focusing on the *ERBB2* copy numbers in the paired samples, we observed a trend of decreasing *ERBB2* copy numbers in post-T-DXd samples compared to pre-T-DXd samples; however, cautions should be taken for this trend might have stemmed from the technical limitations of targeted sequencing or the discrepancy in tumor purity in each sample. Taken together, we found that the overall mutational landscapes of pre-T-DXd and post-T-DXd human samples are largely similar; however, certain discrepancies do exist that necessitate additional study in a larger cohort of T-DXd-treated population.

RNA-seq was conducted on the paired samples from PT01, PT02, and PT03. To elucidate the enriched cellular pathways in pre- and post-T-DXd treatment BC samples, we performed GSEA on three paired samples. Most importantly, MYC_TARGETS_V2 (normalized enrichment score [NES] = 2.144; FDR q-value < 0.001), MTORC1_SIGNALING (NES = 2.134; FDR q-value = 0.002), and DNA_REPAIR (NES = 1.625; FDR q-value = 0.002) were significantly enriched in post-T-DXd samples (Fig. 1D, Sup. Table 7).

### HER2-directed ADC-resistant HER2+ BC cell lines show reduced *ERBB2* gene and HER2 protein expression

To better understand the mechanisms of the molecular changes in patient samples after the development of resistance to HER2-directed ADC therapies, we established HER2-directed ADC–resistant HER2+ BC cell lines. First, we determined the *ERBB2* gene copy number in five HER2+ BC cell lines: SKBR3, BT474, HCC1954, SUM190, and HCC1419. We confirmed that all tested HER2+ BC cell lines were *ERBB2* gene amplified (*ERBB2* gene copy number: > 10) compared to the triple-negative BC cell line MDA-MB-231 that was known to HER2-negative cell line (*ERBB2* gene copy number: 1.01) (Fig. S1A). Next, we evaluated the sensitivity of HER2+ BC cell lines to HER2-ADC and observed a dose-dependent response to T-DM1 or T-DXd treatment in the 5-day short-term treatment condition (Fig. S1B).

On the basis of proliferation data, we selected SUM190 (which had the highest HER2 copy number) and HCC1954 (which had the lowest) (Fig. S1A). Before generating HER2-directed ADC-resistant cell lines, we first tested the antitumor effects of T-DM1 and T-DXd in xenograft models and confirmed that single-agent treatment led to tumor shrinkage in both SUM190 (-66.84% GI and -25.56% GI respectively, *P* < 0.001) and HCC1954 (-18.60% GI and -22.82% GI respectively, *P* < 0.05) xenografts compared to in vehicle control (Fig. S1C).

**Fig. 2:**
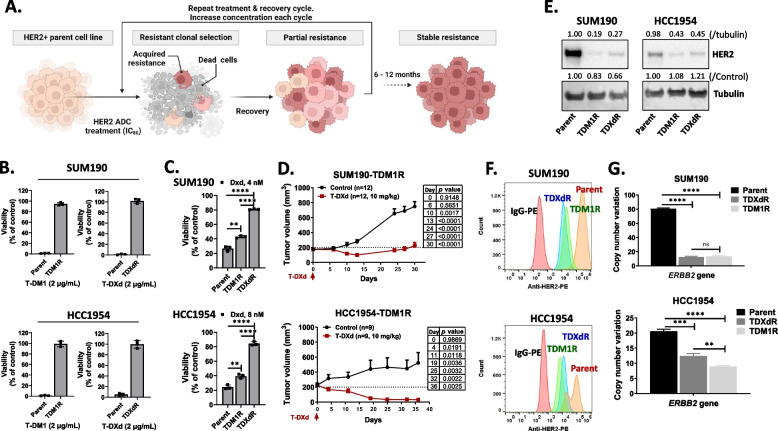
Anti-HER2 antibody–drug conjugate (HER2-directed ADC)–resistant HER2+ BC cell line generation. **A** TDM1R and TDXdR cell lines generated by continuous treatment/recovery cycle with HER2-directed ADC. SUM190 (1 million) and HCC1954 (500,000) cells were added to the 100-mm culture dish. The next day, cells were treated with T-DM1 or T-DXd at the 80% inhibitory concentration (IC_80_) for 3–5 days and then replaced with fresh complete media until cells recovered at a normal growth rate. This treatment/recovery cycle was repeated for about 6–12 months. **B** Clonogenic assay. Parent TDM1R and TDXdR cell lines were treated with 2 µg/ml of T-DM1 or T-DXd for 14 days, and viability was measured by an SRB staining assay. Experiments were repeated three times independently. Data were collected from three biological replicates. **C** Antiproliferation effect of T-DXd payload and DXd in parent and TDM1R and TDXdR cell lines. Cells were treated with DXd for 14 days, and viability was measured by the SRB staining assay. Data were collected from three biological replicates. **D** T-DXd significantly reduces tumor growth in SUM190-TDM1R (*n* = 12 per group) and HCC1954-TDM1R (*n* = 9 per group) xenograft models. A multiple *t*-test comparison was used to compare tumor size between the control and treatment groups. **E** TDM1R and TDXdR cell lines showed reduced HER2 expression. The ImageJ program was used to measure intensity. Western blotting. **F** FACS analysis. TDM1R and TDXdR cell lines showed reduced cell-surface HER2 expression. Cells were maintained without drug for 7 days and collected to measure HER2 expression on the cell surface with anti-HER2-PE. Three biological replicates showed similar results.** G** Droplet digital PCR assay. CNV indicates copy number variation. TDM1R and TDXdR cell lines showed a reduced *ERBB2* gene copy number. Each box shows mean with standard deviation; **, *P* < 0.01; ***, *P* < 0.001, ****, *P* < 0.0001, n.s. not significant. Data were collected from three biological replicates

To generate HER2-directed ADC–resistant BC cell lines, we treated SUM190 and HCC1954 parent cells with T-DM1 or T-DXd at the 80% inhibitory concentration (IC_80_) for 3–5 days and then replaced the culture media with fresh complete media until cells recovered at a normal growth rate. This treatment/recovery cycle was repeated for about 6–12 months (Fig. 2A). We confirmed that no TDM1R and TDXdR HER2+ BC cell lines showed growth inhibition when treated with 2 µg/mL of T-DM1 or T-DXd while parent BC cell lines showed over 90% cell death with the same treatment (Fig. 2B; TDM1R and TDXdR indicate resistance to TDM1 and T-DXd, respectively). We also confirmed that both SUM190-TDXdR and HCC1954-TDXdR cell lines did not show growth inhibition when treated with the T-DXd payload, DXd(*P* < 0.0001, Fig. 2C) but did show growth inhibition in TDM1R-resistant cell lines (*P* < 0.01, Fig. 2C).

To confirm the antitumor effect of T-DXd in TDM1R BC models, we conducted a xenograft assay and confirmed that T-DXd significantly reduced tumor growth in both SUM190-TDM1R (70.5% GI) and HCC1954-TDM1R (-85.9% GI) xenograft models (Fig. 2D, *P*< 0.01). Next, we determined the HER2 expression levels in TDM1R and TDXdR BC cell lines using Western blotting and fluorescence-activated cell sorting (FACS) analysis. Both T-DM1- and T-DXd-resistant HER2+ BC cell lines had reduced total HER2 protein and cell surface HER2 expression levels (Fig. 2E and F). To determine whether reduced HER2 protein expression in TDM1R and TDXdR BC cell lines was due to downregulation of gene expression levels, we performed a droplet digital PCR assay and observed reduction of *ERBB2* gene copy numbers in all TDM1R and TDXdR BC cell lines (SUM190-TDM1R and SUM190-TDXdR: > 80% reduction, *P* < 0.0001, SUM190-TDM1R and SUM190-TDXdR: > 40% reduction, *P* < 0.001) compared to in parent cell lines (Fig. 2G).

### Genetic alterations cause downregulation of HER2 gene expression

**Fig. 3:**
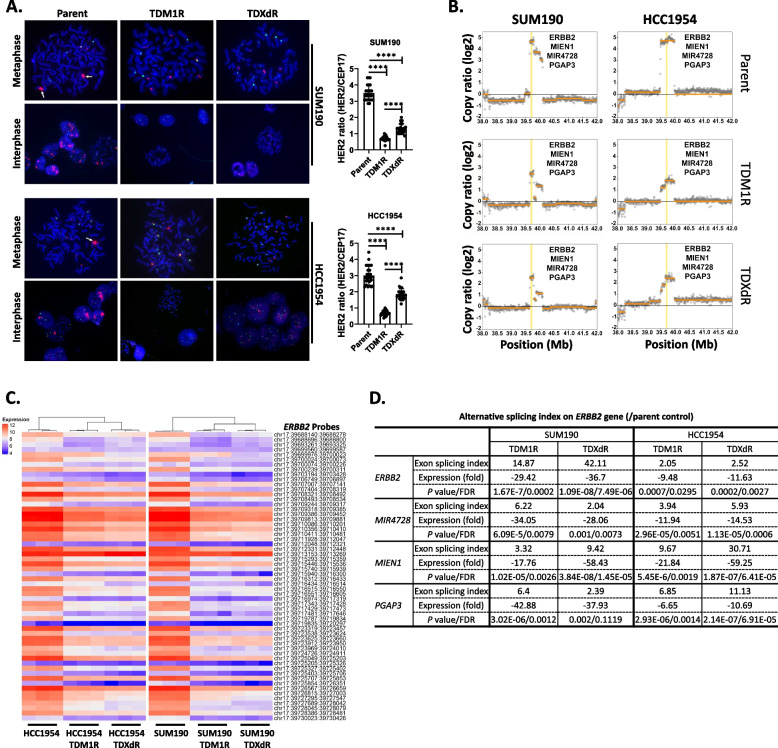
HER2-directed ADC-resistant HER2+ BC cell lines showed reduced *ERBB2* gene amplification. **A** Fluorescence in situ hybridization analysis. The red color indicates the amplification of *ERBB2* gene, and the green color indicates the centromere on chromosome 17 (CEP17). A total of 25 individual cells were evaluated for ERBB2 gene amplification by measuring the HER2/CEP17 ratio from each cell line. Each box shows mean ± s.d.; ****, *P* < 0.0001. Three biological replicated experiments showed similar results. **B** Whole-genome sequencing data analysis. *ERBB2, MIEN1, MIR4728,* and *PGAP3* gene copy numbers were reduced on chromosome 17 in TDM1R and TDXdR cell lines. **C** Transcriptome analysis of *ERBB2* gene. All *ERBB2* probes were pulled out from Affymetrix Clariom D Human microarray data and clustered by differential expression. Expression indicates log2. Data were collected from three biological replicates. **D** Alternative splicing was increased in *ERBB2, MIEN1, MIR4728,* and *PGAP3* genes on chromosome 17 compared to in parent cells. Transcriptome Analysis Console (TAC, Affymetrix, Inc) software used for the Affymetrix Clariom D Human microarray database to compare differential gene splicing between HER2-ADC-resistant cells and their parent cells. Data were collected from three biological replicates

TDM1R and TDXdR BC cell lines showed reduced HER2 gene copy number and protein levels (Fig. 2). We validated the HER2 gene copy number data by fluorescence in situ hybridization analysis using the T-DM1R and TDXdR BC cell lines. As shown in Fig. 3A, parent SUM190 and HCC1954 cell lines showed HER2 gene amplification (HER2/CER17 ratio: 3.497 and 3.002, respectively), but SUM190-TDM1R (0.7004), SUM190-TDXdR (0.1352), HCC1954-TDM1R (0.6826), and HCC1954-TDXdR (1.851) cell lines showed less HER2 gene amplification in both metaphase and interphase than did parent cells (*P* < 0.0001). To elucidate how HER2-directed ADC reduces HER2 gene expression in HER2+ BC cell lines, we first assessed chromosome instability, which is closely related to cancer development, gain or loss of gene expression, and therapeutic resistance [33, 34]. We analyzed chromosomes in 35 metaphase cells from each parent cell line and TDM1R and TDXdR BC cell lines. We observed chromosomal aberrations in both parent and TDM1R and TDXdR BC cell lines; however, these aberrations were not increased in TDM1R and TDXdR BC cell lines compared to in parent cells (Fig. S2A). Interestingly, the SUM190-TDXdR cell line showed truncation of the HER2 gene-amplified region (Fig. S2B). On the basis of this observation, we analyzed the gene copy number ratios between the parent and TDM1R and TDXdR BC cell lines of the gene copy numbers of *ERBB2*, *MIEN1*, *MIR4728*, and *PGAP3* near the HER2 gene location, using whole-genome sequencing data. In the SUM190-TDM1R and SUM190-TDXdR cell lines, the gene copy numbers of *ERBB2, MIEN1, MIR4728,* and *PGAP3* were reduced by at least 50% compared to the SUM190 parent cell line. We observed similar reductions in copy numbers of these genes in the HCC1954 cell lines (Fig. 3B). Interestingly, KPL4, which is relatively resistant to T-DXd compared to SUM190 (> tenfold higher than IC_50_ of SUM190) and chronically exposed to T-DXd with 2 µg/ml (KPL4-TDXdR), retained HER2 gene expression, but the T-DM1-resistant KPL4 cell line showed reduced HER2 gene copy numbers (Fig. S2C). Altogether, these data suggest that chronic exposure to the HER2-ADCpayload altered HER2 gene expression in TDM1R and TDXdR HER2+ BC cell lines.

Alternative splicing is also a well-known mechanism for regulating gene expression [35]. To measure the alternative splicing of HER2 mRNA in TDM1R and TDXdR BC cell lines, we first checked the binding intensity of all HER2 gene probes using the Clariom D human microarray chip. Gene expression data indicated that 93% (54 of 58) of HER2 gene probes showed reduced HER2 gene expression in both TDM1R and TDXdR BC cell lines compared to in parent cell lines (Fig. 3C), and we confirmed that the alternative splicing index of *ERBB2*, *MIEN1*, *MIR4728*, and *PGAP3* genes was significantly increased in both types of TDM1R and TDXdR BC cell lines, resulting in mRNA reduction (Fig. 3D). Taken together, our data indicated that ADC-mediated genetic alterations such as deletion, reduction of gene copy number, and alternative splicing cause downregulation of HER2 gene expression in HER2+ BC cell lines.

There are four major potential mechanisms of resistance against ADC: 1) reduced ADC uptake due to the reduction of the target molecule, 2) efflux of the payload, 3) epigenetic modification, and 4) bypass of the payload’s antitumor effect by activation of signaling pathways [36, 37]. The mechanism of target protein reduction appears to be at play in our observation of reduced HER2 expression in TDM1R and TDXdR cell lines. To confirm whether reduced HER2 protein level is the reason for T-DXd resistance, we overexpressed HER2 in TDXdR BC cell lines and measured the antiproliferation effect of T-DXd. Overexpression of HER2 did not induce the antiproliferation effect of T-DXd (Fig. S3A). To show that reduced HER2 expression in TDM1R and TDXdR cell lines is sufficient for ADC uptake and therapeutic efficacy, we evaluated the antitumor effect of T-DXd in SUM190-TDM1R and HCC1954-TDM1R xenograft models, which have reduced HER2 expression (Fig. 2D and Fig. S8C). T-DXd significantly reduced tumor growth in both SUM190-TDM1R and HCC1954-TDM1R xenograft models (Fig. 2D, *P*< 0.01). These data indicated that reduced HER2 expression is not a major cause of resistance to HER2-ADC, and reduced HER2 expression in TDM1R and TDXdR BC cell lines is still sufficient to induce tumor growth inhibition by HER2-directed ADC endocytosis.

We did not observe a change in multidrug-resistant genes such as *MDR1* and *ABCG2* on the microarray analysis, but the epigenetic modulator *EGR1* and carrier protein gene *SLC6A14* were significantly elevated in TDM1R and TDXdR BC cell lines. To validate whether *EGR1* or *SLC6A14* is involved in HER2-directed ADC resistance, we knocked them down using RNAi in DXd-resistant BC cell lines and performed a proliferation assay with T-DXd. Silencing of *EGR1* or *SLC6A14* did not increase the efficacy of T-DXd in SUM190-TDXdR and HCC1954-TDXdR cell lines (Fig. S3B). These data indicated that HER2-directed ADC resistance is not caused by a reduction in intracellular DXd payload level or HER2 expression.

### DNA damage response pathway can be targeted to enhance the antitumor effect of T-DXd in TDM1R and TDXdR HER2+ BC cell lines

**Fig. 4:**
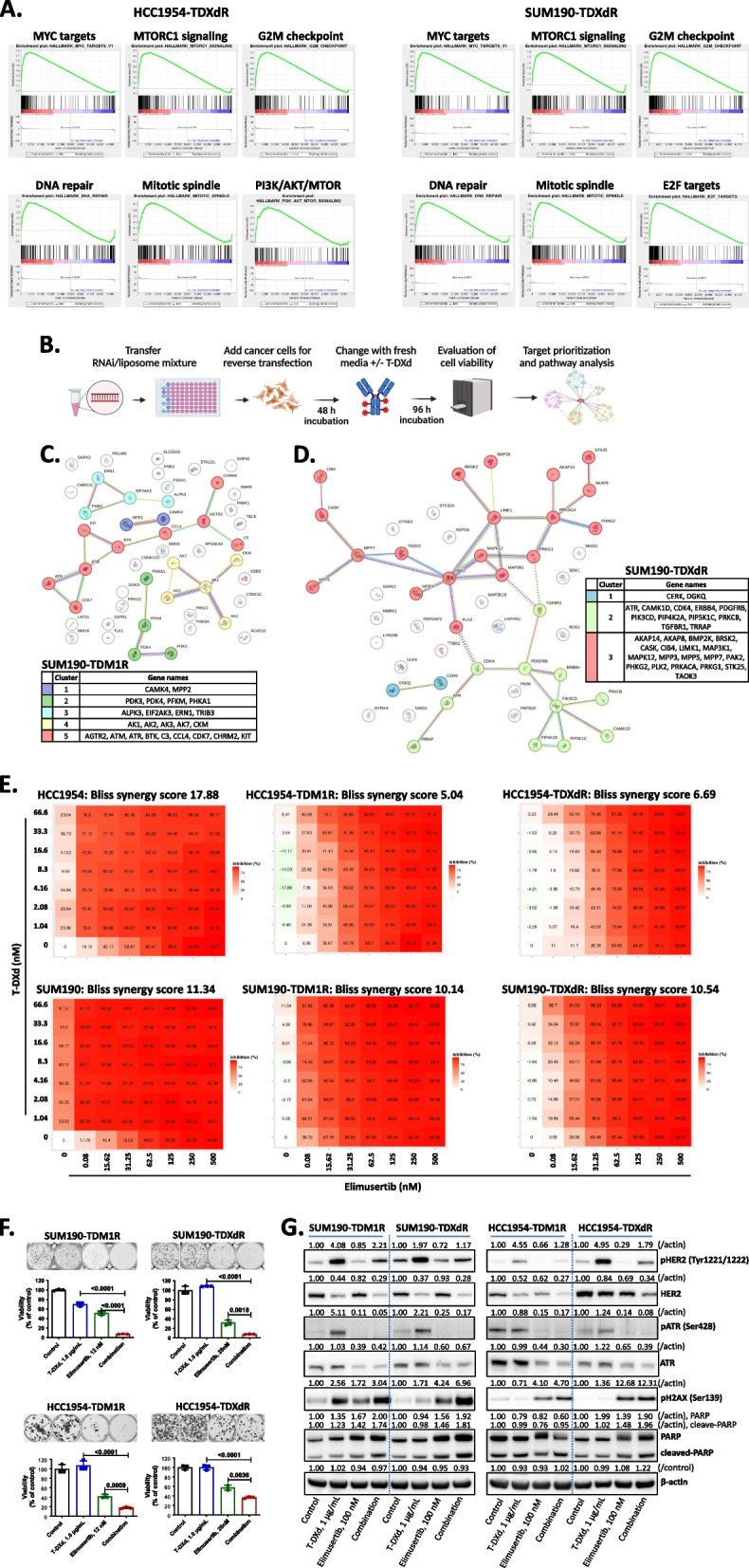
A gene expression analysis and synthetic lethal kinome library high-throughput RNAi screening revealed that the DNA repair pathway is a target for enhancing the efficacy of T-DXd in TDM1R and TDXdR cell lines. **A** Functional gene-set enrichment analysis using Affymetrix Clariom D Human Transcriptome array data. **B** Illustration of synthetic lethal kinome library high-throughput RNAi screening. **C** and **D** STRING interaction analysis of the top 50 target genes from kinome library high-throughput RNAi screening. K-means clustering was applied to identify groups of genes with similar behavior. Each color indicated co-regulated gene modules related to a specific canonical signaling pathway. SUM190-TDM1R (C), SUM190-TDXdR (D). **E** Bliss independence dose–response assay. Cells were treated with T-DXd and elimusertib for 5 days, and viability was measured using SRB staining. The data shown are representative of three independent experiments with similar results—the table indicates viability, and the Bliss synergy score was evaluated and visualized using Synergyfinderplus software (right, www.synergyfinderplus.org). **F** Clonogenic assay. Cells were treated with T-DXd and/or elimusertib for 14 days, and cell viability was measured by SRB staining. Data are presented as mean ± standard deviation. Two-tailed unpaired Student’s *t*-test. Experiments were repeated in triplicate**. G**. Western blotting. Cells were treated with T-DXd (1 µg/ml) and/or elimusertib (100 nM) for 48 h, and whole-cell lysates were collected for immunoblotting. Protein expression was normalized with actin level in control cells from each TDM1R and TDXdR cell line using ImageJ software. The data shown are representative of three independent experiments with similar results

We confirmed the presence of *ERBB2* gene alterations in patients with HER2+ BC after anti-HER2 therapy and in TDM1R and TDXdR HER2+ BC cell lines. Both payloads, DM1 and DXd, are well known to induce DNA damage response pathways due to increased genotoxic stress by inhibition of DNA replication, transcription, recombination, and chromatin remodeling. Hence, we hypothesized that the DNA damage response pathway can be targeted to overcome the resistance to HER2-directed ADCs. We analyzed microarray data from TDM1R and TDXdR BC cell lines using Transcriptome Analysis Console Software to identify canonical pathways and found that DNA damage response was activated in T-DM1R and TDXdR BC cell lines (Fig. S4A). To confirm these findings, we also performed a gene set enrichment analysis and found that a DNA repair, G2M checkpoint, and mitotic spindle pathways were activated in the TDXdR cell lines (Fig. 4A and Sup. Table 8), which are known to regulate mitosis. In TDM1R BC cell lines, we only observed an activated DNA damage response pathway in SUM190-TDM1R cell line (Sup. Table 9). A further signaling network analysis showed that DNA repair pathway genes—such as non-homologous end joining, mismatch repair, nucleotide excision repair, base excision repair, and homologous recombination repair pathway–related genes—were expressed in TDM1R and TDXdR BC cell lines (Fig. S4B-E). We further determined the expression level of DNA repair pathway proteins using a reverse-phase protein array database and observed up-regulation of ATR, pATR, ATM, Chk1, Chk2, Rad50, and Rad51 in HER2-ADC-resistant cell lines compared to its parent cells (Fig. S4F-H). These data support our hypothesis that DNA damage response pathway inhibition could enhance the efficacy of HER2-directed ADC in HER2-directed ADC–resistant HER2+ BC.

We identified potential kinase targets whose inhibition could overcome HER2-directed ADC resistance or enhance the antitumor efficacy of HER2-directed ADC in TDM1R and TDXdR BC cell lines. We focused on T-DXd for the synthetic lethal screening because recent clinical trial data indicated that T-DXd is associated with improved overall survival and progression-free survival compared to T-DM1 in patients with HER2+ metastatic BC [38]; T-DXd is also now a standard second-line therapy in these patients. We performed a non-biased high-throughput RNAi screening in SUM190-TDM1R and SUM190-TDXdR cells using kinome library pooled siRNA, consisting of 2,127 siRNAs targeting 709 kinase genes (Fig. 4B). Using the Sensitivity Score analysis (described in supplemental information), we selected top 50 target kinases from each of the SUM190-TDM1R and SUM190-TDXdR cell lines (Sup. Table 10). A STRING interactome pathway analysis identified DNA repair pathway–related genes as potential targets for combination therapy with T-DXd in both TDM1R and TDXdR BC cell lines. Ataxia-telangiesctasia, mutated (ATM), ataxia telangiectasia and Rad3-related (ATR), and CDK7 were identified in the SUM190-TDM1R cell line (Fig. 4C), and ATR was identified in SUM190-TDXdR cell line (Fig. 4D). Specifically, ATR was overlapped in both cell lines. Taken together, the patient data analysis and synthetic lethal screening data indicated that the DNA repair pathway is a potential target whose inhibition could enhance the efficacy of T-DXd in TDM1R and TDXdR BC cell lines. To verify the RNAi screening result, we tested two additional RNAi targeting ATR and evaluated its synergistic antiproliferation effect with T-DXd in HER2 ADC-resistant HER2+ BC cell lines. We observed that two ATR RNAi significantly inhibited growth and showed a combination antiproliferation effect with T-DXd in tested all HER2+ BC cell lines (*P* < 0.05, Fig. S5A and S5B).

### ATR inhibitor elimusertib enhanced the antitumor efficacy of T-DXd in TDM1R and TDXdR HER2+ BC xenograft models

Patients’ gene alteration and gene expression data (Fig. 1), microarray data in TDM1R and TDXdR cell lines (Fig. 4A and Fig. S4A), and kinome RNAi screening results (Fig. 4C and D) indicated that the PI3K, cell cycle and DNA repair pathways are potential targets for combination therapy with T-DXd in TDM1R and TDXdR HER2+ BC. To validate which target can enhance the efficacy of T-DXd in TDM1R and TDXdR cell lines, we selected specific kinase inhibitors against PI3K (alpelisib), CDK4/6 (abemaciclib), Wee1 (AZD1775), Aurora kinase A (TAS-119), ATR (elimusertib), and PARP inhibitor (olaparib) for proliferation assays. We limited drugs that were only FDA-approved or had been used for clinical trials. As single agents, abemaciclib and elimusertib showed significant growth inhibition in all tested TDM1R and TDXdR cell lines (> 60% GI), but alpelisib, AZD1775, TAS-119, and olaparib did not (Fig. S5C and S5D). In combination with T-DXd, we found that only the ATR inhibitor elimusertib showed an enhanced antiproliferation effect in both T-DM1R and TDXdR BC cell lines (Fig. S5C and S5D). To evaluate the combination effect of T-DXd and elimusertib, we conducted a Bliss independence dose–response surface model [39, 40] under the 5-day short-term treatment condition. Compared to T-DXd or elimusertib monotherapy, combination therapy significantly inhibited antiproliferation in all tested TDM1R and TDXdR cell lines (Bliss synergy score: 11.34 in SUM190; 10.54 in SUM190-TDM1R; 10.14 in SUM190-TDXdR; 17.88 in HCC1954; 5.04 in HCC1954-TDM1R; 6.68 in HCC1954-TDXdR) (Fig. 4E, Sup. Figure 5E and 5F). Additionally, we tested small molecules Gartisertib (ATR inhibitor) and AZD1390 (ATM inhibitor) to confirm the target specificity of the DNA repair pathway. Similar to elimusertib and T-DXd combination data, we observed an enhanced antiproliferation effect in combination treatment in HER2 ADC-resistant SUM190 and HCC1954 cell lines (Fig. S5G and S5H). These data indicated that the DNA repair pathway, particularly ATR, is a potential target for combination with T-DXd in TDM1R and TDXdR HER2+ BC.

Next, we examined the combination effect of T-DXd and elimusertib on long-term treatment conditions using a clonogenic assay. Compared to T-DXd or elimusertib monotherapy, combination therapy significantly inhibited antiproliferation in all tested TDM1R and TDXdR cell lines (*P* < 0.0001 in SUM190-TDM1R; *P* < 0.01 in SUM190-TDXdR; *P* < 0.001 in HCC1954-TDM1R; *P* < 0.01 in HCC1954-TDXdR) (Fig. 4F).

We next determined whether the enhanced antiproliferation effect of the combination treatment was a result of apoptosis induction by ATR inhibition. In the SUM190-TDM1R and SUM190-DXdR cell lines, the combination of T-DXd and elimusertib induced DNA damage marker pH2AX and cleaved PARP expression compared to single-agent treatment with T-DXd or elimusertib (Fig. 4G, left panel, and Fig. S6A). In HCC1954-TDM1R cells, elimusertib single-agent treatment significantly induced pH2AX. The combination of T-DXd and elimusertib did not induce an increase in the apoptosis marker cleaved PARP but did result in reduced full-length PARP expression compared to single-agent T-DXd and elimusertib. We speculate that the apoptosis pathway activated before the 48-h time point. In HCC1954-TDXdR cells, we observed enhanced cleaved PARP expression upon combination treatment, but pH2AX expression was not increased by the combination treatment compared to single-agent elimusertib (Fig. 4G, right panel, and Fig. S6A). Unlike the reduction of HER2 expression by T-DXd treatment, pHER2 expression was significantly increased by T-DXd in all tested cell lines (Fig. 4G).

We determined whether elevated pHER expression affects its downstream molecules. We first analyzed a reverse-phase protein array database and observed downregulation of HER2 and upregulation of pHER2 expression with T-DXd treatment; however, we did not observe significantly increased expression of key downstream molecules, such as pAKT, pmTOR, pS6K, p70S6K, pMEK1, and pMAPK (pERK) (Fig. S6B). Further, we validated the reverse-phase protein array data using Western blotting and confirmed that T-DXd-mediated pHER2 induction does not induce its downstream molecules, pAKT and pERK (Fig. S6C and S6D).

**Fig. 5:**
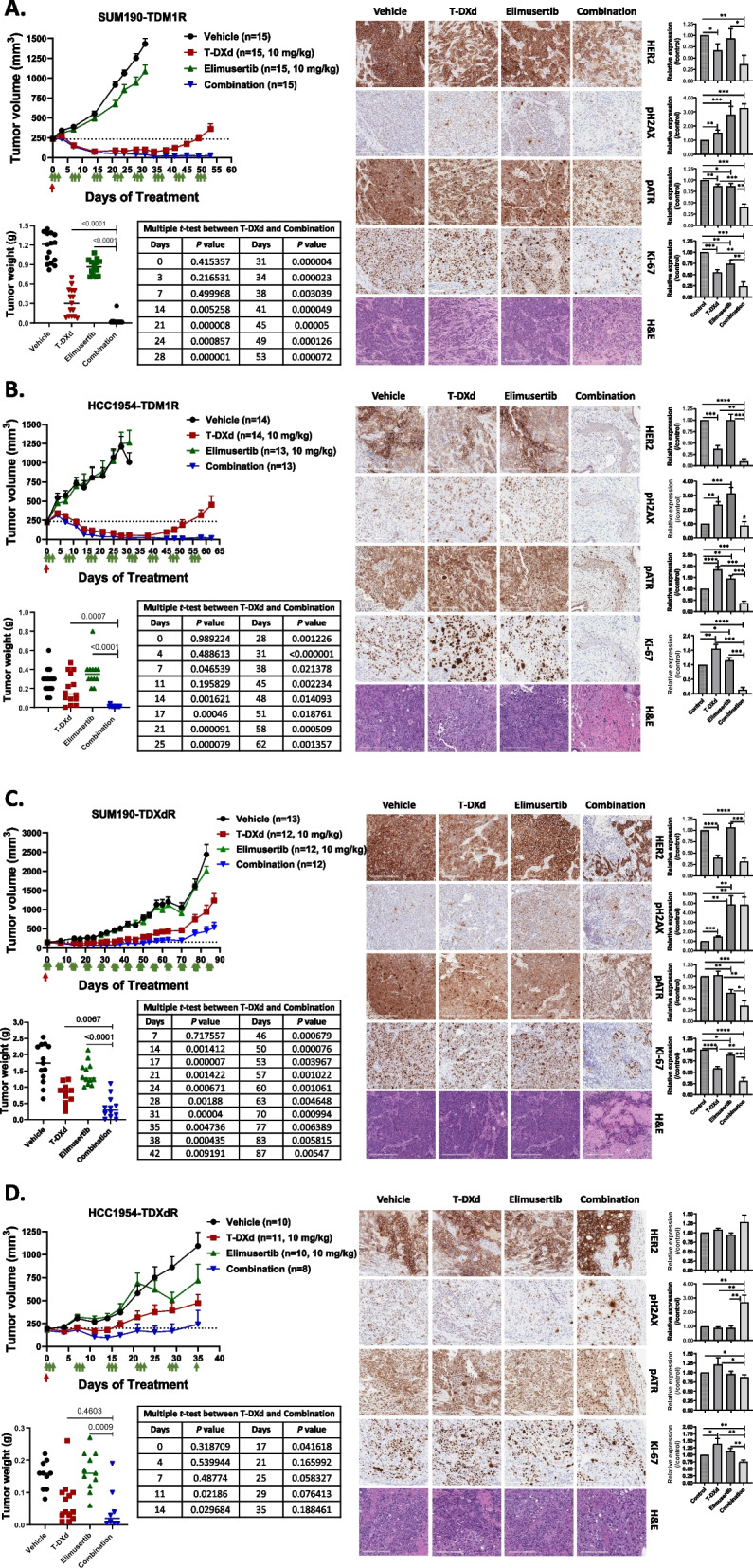
Combination treatment with T-DXd and elimusertib enhanced the antitumor effect compared with monotherapy in TDM1R and TDXdR HER2+ BC in vitro and in vivo. **A-D** Xenograft assay using SUM190-TDM1R (**A**), HCC1954-TDM1R (**B**), SUM190-TDXdR (**C**), and HCC1954-TDXdR (**D**). Cells were injected into the mammary fat pad of nude mice, and treatments were started when tumors were an average of 200—250 mm^3^. T-DXd (10 mg/kg) was administered one time on Day 0 via tail-vein injection. Elimusertib (10 mg/kg) was administered via oral gavage twice a day (6-h intervals) for 3 consecutive days per week. Data are presented as mean ± standard deviation. *Left*, tumor growth and tumor weight (endpoint) measurements. Table shows multiple *t*-tests between T-DXd and combination on each measurement date. *Right*, IHC images of expression levels of HER2, pH2AX, pATR, and Ki-67 in xenograft tumor tissues. Multiple *t*-test comparison tests were used for tumor growth. Table shows *t*-tests between T-DXd and a combination of elimusertib and T-DXd on each measurement date. A two-tailed unpaired Student’s *t*-test was used for tumor weight comparison. Scale bars = 200 µm. IHC intensity was evaluated using the ImageJ program. Each box shows the mean with standard deviation. * *P* < 0.05, ** *P* < 0.01, *** *P* < 0.001, **** *P* < 0.0001. The data shown are representative of three tumor samples per group with similar results

Our in vitro data demonstrated that ATR inhibition enhances the efficacy of T-DXd in HER2-directed, ADC-resistant HER2+ BC cells. Next, we examined the synergistic antitumor effect of T-DXd and elimusertib in SUM190-TDM1R, SUM190-TDXdR, HCC1954-TDM1R, and HCC1954-TDXdR xenograft models. The doses were 10 mg/kg for T-DXd [7] and 10 mg/kg for elimusertib [41]. Compared to vehicle control, single-agent T-DXd treatment showed significant tumor shrinkage in both SUM190-TDM1R (74% shrinkage, *P* < 0.0001) and HCC1954-TDM1R (75% shrinkage, *P* < 0.0001) xenograft models until days 35–40, when recurrence occurred (Fig. 5A and B). Elimusertib single-agent treatment did not have an antitumor effect but showed a synergistic antitumor effect with T-DXd, and this combination showed more sustained tumor shrinkage than did T-DXd single-agent treatment in both the SUM190-TDM1R (*P* < 0.0086) and HCC1954-TDM1R (*P* < 0.0383) models (Fig. 5A and B). After the completion of combination treatment, some mice showed no residual tumors (3 of 13 mice with SUM190-TDM1R and 4 of 12 mice with HCC1954-TDM1R). In the SUM190-TDXdR xenograft model, single-agent T-DXd treatment did not show tumor shrinkage but rather continual tumor growth. As in the T-DM1-resistant model, single-agent elimusertib treatment did not show tumor growth inhibition (TGI); however, elimusertib combined with T-DXd showed a significant TGI effect compared to single-agent T-DXd in the SUM190-TDXdR model (Fig. 5C, 57% TGI, *P* < 0.0305). In the HCC1954-TDXdR xenograft model, we did not observe significant TGI upon combination treatment with T-DXd and elimusertib compared to single-agent T-DXd (Fig. 5D, 33% TGI, *P* < 0.5795). However, when we performed a paired comparison analysis, only three mice bearing HCC1954-TDXdR xenografts treated with the combination showed tumor progression; the remaining five mice showed tumor shrinkage or growth inhibition in the combination treatment group.

We analyzed the expression levels of HER2, Ki-67, pATR, and pH2AX in xenograft tissues by IHC staining as observed in the Western blotting data (Fig. 4G), the expression levels of HER2, pATR, and Ki-67 were reduced in tumors from SUM190-TDM1R and HCC1954-TDM1R xenograft models treated with T-DXd combined with elimusertib compared to single-agent treatment with T-DXd or elimusertib (Fig. 5A and B). In tumors from SUM190-TDXdR and HCC1954-TDXdR models, we observed only the inhibition of proliferation marker Ki-67 by T-DXd combined with elimusertib (Fig. 5C and D). We speculate that a one-time injection of T-DXd is not sufficient to enhance tumor cell death on long-term follow-up because both SUM190-TDXdR and HCC1954-TDXdR xenograft models showed recurrence in all groups. There was no body weight loss in the mice treated with T-DXd, elimusertib, or the combination during the treatment period (Fig. S7), supporting the safety of the tested T-DXd and elimusertib treatment dose and schedule. Taken together, these results suggest that the ATR inhibitor elimusertib enhances the antitumor effect of T-DXd via the DNA damage pathway in both T-DM1- and T-DXd-resistant HER2+ BC. The IHC results demonstrated strong HER2 expression in all TDM1R and TDXdR BC xenograft models. The drug treatment started between 14 to 21 days after cell inoculation into mice. To confirm whether HER2 expression was restored during tumor engraftment in mice, we stopped T-DM1 and T-DXd treatment in all cell lines for 1 month and measured total and cell surface HER2 expression levels by Western blotting and FACS analysis, respectively (Fig. S8A and S8B), as well as IHC staining in SUM190 and HCC1954 parent tissue samples (Fig. S8C). We confirmed that HER2 levels were continually downregulated in TDM1R and TDXdR BC in cell lines and xenografts. These data indicated that the enhanced HER2 signals were caused by detection conditions without parent tissue samples.

## Discussion

In the management of HER2+ BC, FDA-approved therapies such as T-DM1 and T-DXd have markedly improved survival rates. However, resistance to HER2-directed ADCs remains a challenge, with no established therapy post-resistance to T-DXd. Our translational research underscores the importance of the DNA repair pathway in this resistance, identifying it as a potential therapeutic target. We discovered that chronic exposure to T-DM1 or T-DXd reduces HER2 expression and induces oncogenic transformation such as copy number variation, gene amplifications, and epigenetic modifications. Further, enhanced DNA repair activity post-ADC treatment in patient samples suggests new intervention opportunities. Additionally, using a synthetic lethal kinome library RNAi screen informed by patient data, in non-biased manner, we identified targets that significantly increase T-DXd's effectiveness. Our preclinical models show that combining T-DXd with elimusertib, an ATR inhibitor, is a promising strategy to overcome resistance. These results support integrating DNA repair inhibitors into the therapeutic repertoire for HER2 ADC-resistant BC tumors, thus representing a significant advance in treatment protocols.

Although tremendous progress has been achieved with targeted therapy for HER2+ BC, these tumors eventually develop resistance. A better understanding of the mechanisms of resistance to anti-HER2 therapy is needed to develop new therapeutic approaches. While several mechanisms of resistance to trastuzumab or T-DM1 have been described, there is no comprehensive analysis identifying the mechanisms of resistance to T-DXd in HER2+ BC, but it is expected that some mechanisms described for trastuzumab and/or T-DM1 can be extrapolated to T-DXd. Alterations in the *ERBB2* gene are considered a representative mechanism of resistance to anti-HER2 therapies. In fact, our current study showed frequent HER2 loss in models of resistance to trastuzumab and pertuzumab or T-DM1. Since T-DM1 and T-DXd are ADCs directed against HER2, HER2 loss or decreased HER2 expression is a possible cause of resistance [42, 43]. A recent study affirmed HER2 status changes in metastatic HER2+ BC after treatment, and patients with loss of HER2 showed worse responses to T-DM1 and inferior overall survival [44]. In the KRISTINE trial, a phase III trial of neoadjuvant T-DM1 and pertuzumab in HER2+ BC, a subgroup of 15 patients treated with T-DM1 and pertuzumab who had locoregional progression before surgery showed high heterogeneity in HER2 expression, which may have contributed to the worse clinical outcomes observed with T-DM1 treatment [45]. In vitro, several cell lines generated with acquired resistance to T-DM1 showed a decrease in HER2 expression compared to parent cells [46–50].

Recently, HER2-directed ADC has been used for targeted systemic delivery of chemotherapeutic agents as payload, such as DM1, DXd, duocarmycin, monomethyl auristatin F, and monomethyl auristatin E, to inhibit DNA replication. Due to the mechanism of action of the payload, chemotherapeutic agents cause DNA alterations, including mismatches, single-strand breaks, and double-strand breaks, resulting in gene mutation and genome instability [51]. Thus, cancer cells activate the DNA damage response to escape chemotherapeutic agent–mediated cell death. To overcome the drug resistance related to the DNA repair pathway, inhibitors of ATM, ATR, CHK1, Wee1, DNA-PK, and PARP are potential candidates for enhancing these chemotherapies and are currently in clinical trials in combination with other anticancer drugs. ATM mutations have been associated with an increased risk of BC [52, 53]. A preclinical study indicated that Wee1 inhibitor use has potential clinical applications in overcoming trastuzumab resistance in BC [54]. Also, the PARP inhibitor olaparib showed a synergistic antitumor effect with T-DXd in HER2+ and HER2-low BC xenograft models [55].

In the phase 2 DAISY trial (ClinicalTrials.gov identifier: NCT04704661) [56], the authors found that SLX4 loss of function mutation is absorbed in T-DXd-resistant HER2+ BC patients and has a role in the T-DXd resistance mechanism in BC cell lines. In our study, we investigated HER2 and other gene alterations in patient samples and cell lines after the development of HER2-directed ADC resistance and found alterations of genes related to the DNA repair pathway. We did not observe SLX4 mutation in five matched pre- and post-T-DXd treatment tissue samples or five post-T-DXd treatment samples. Indeed, WGS data from SUM190 parent, SUM190-TDM1R, and SUM190-TDXdR cell lines showed SLX4 mutation, but HCC1954 cell lines showed no changes in SLX4 mutation (Fig. S9). Unlike in other studies, SUM190 cells were more sensitive to T-DXd treatment than were other HER2+ cell lines. These data implied that SLX4 is not a single driver in resistance to T-DXd. Thus, further investigations are needed to elucidate the function of SLX4 mutation.

Increasing studies have found that drug resistance in cancer cells is closely tied to the DNA repair regulatory system. In HER2+ cancers, there is an ongoing phase I/IB clinical trial of the combination of T-DXd and an ATR inhibitor in patients with advanced solid tumors expressing the HER2 protein or gene (DASH trial, ClinicalTrials.gov identifier: NCT04704661). ADCs are designed to elicit a tumor-selective therapeutic effect; however, some adverse effects are considered a clinical obstacle to developing combination regimens with DNA repair pathway–targeting inhibitors. The most common adverse effects of HER2-directed ADC are fever, nausea, vomiting, and hematologic toxicity [57, 58]. In the DESTINY-Breast01 trial, T-DXd was associated with leukopenia, anemia, fatigue, nausea, interstitial lung disease, and neutropenia [9]. The ATR inhibitor elimusertib has antitumor activity in advanced solid tumors, including BC [41, 59]. The most common treatment-emergent adverse events were generally hematologic and comprised anemia (81.8%, grade 3), neutropenia (72.7%, grade 3/4), and thrombocytopenia (45.5%, grade 3/4). Fatigue (68.2%, grade 2) and nausea (50.0%, grade 3) were also reported (ClinicalTrials.gov identifier: NCT03188965) [59]. Therefore, more severe hematologic adverse events are expected to occur when patients are treated with T-DXd and elimusertib together. To reduce severe hematological adverse events, dose de-escalation or sequential treatment with T-DXd and elimusertib, rather than concomitant treatment, should be considered. The most common concentration of elimusertib used in preclinical xenograft models is 20–50 mg/kg [41, 60]. In our preclinical studies, 10 mg/kg of single-agent elimusertib did not show an antitumor effect, but it enhanced the antitumor effect of T-DXd in TDM1R and TDXdR xenograft models. These data suggest that dose de-escalation of a DNA repair–targeting drug should be considered for the clinical dosing schedule.

Another interesting finding was the significant enrichment of immune-related gene sets in pre-T-DXd samples, including Interferon_Alpha_Response, Interferon_Gamma_Response, and IL6_Jak_Stat3_Signaling (Sup. Table 7). These data indicated an active immune response within the tumor microenvironment in the pre-TDXd treatment group, which turned into an inactive tumor microenvironment after treatment. In the phase 2 DAISY trial, the investigators found that CD68-positive tumor cell-proximate macrophages were significantly decreased after T-DXd treatment [56]. A phase 1b study explored the potential immune system activation benefits of combining atezolizumab and anti-HER2 therapies, including T-DM1, and observed increased PD-L1 levels and CD8 + T-cell infiltration in HER2+ BC [61]. High infiltration of M1-like macrophages and CD8 + T-cells in tumors is associated with better response to trastuzumab therapy in HER2+ BC [62]. Another study observed that high tumor-infiltrated lymphocytes were associated with trastuzumab efficiency and improved survival in BC [63]. Although clinical and preclinical data indicated that the T-DXd and immune checkpoint inhibitor combination [64] is feasible, further investigation is required to elucidate the mechanism of the action of T-DM1 andT-DXd in BC because BC is a heterogenous tumor immune microenvironment due to genetic instability, epigenetic modification, immune cell spatial heterogeneity, and tumor-associated stromal cells such as cancer-associated fibroblasts and adipocytes.

While our study offers valuable insights into the mechanisms of resistance to HER2-directed ADCs and the potential of targeting the DNA repair pathway, it is important to consider certain limitations. Our research primarily uses preclinical models, which may not fully capture the diverse genetic backgrounds found in the HER2+ breast cancer patient population. The variability in cancer resistance mechanisms suggests that further investigation is needed to confirm the efficacy of targeting the DNA repair pathway alongside T-DXd across different cancer types and resistance mechanism. Although we have identified the DNA repair pathway as a promising target, more detailed studies are required to fully understand the mechanisms of resistance and how pathway inhibition enhances T-DXd efficacy. Additionally, expanding the number of patient samples in future studies will be crucial for identifying robust biomarkers, thereby strengthening our approach and enhancing the clinical applicability of our findings.

## Conclusions

In summary, our study identified the induction of HER2 gene alteration and activation of the DNA repair pathway in HER-ADC-resistant BC cell lines and HER2+ BC patients. We validated that the inhibition of the DNA repair pathway can increase sensitivity to T-DXd treatment in TDM-1 resistant and T-DXd resistant BC in vitro and in *vivo*. This preclinical study provides justification for conducting clinical trials with HER2+ BC patients who develop resistance to T-DM1 or T-DXd treatment. We will need for identification of predictive biomarkers that will aid the selection of patients for treatment with T-DXd and the DNA repair pathway–targeted agents.

### Supplementary Information

**Supplementary Material 1:** Supplementary Tables.

**Supplementary Material 2: Supplementary Figure S1.** Antiproliferation effect of T-DM1 and T-DXd in HER2-positive BC cell lines. A. HER2 gene copy variation in HER2+ BC cell lines. The TNBC cell line MDA-MB-231 was used as a negative control. Genomic DNA was used for the ddPCR assay. Each box shows mean with standard deviation. Data were collected from three biological replicates. B. T-DM1 and T-DXd inhibited the proliferation of HER2+ cell lines in a dose-dependent manner. Cells were treated with T-DM1 or T-DXd for 5 days, and viability was measured using SRB staining. The data shown are representative of 3 independent experiments with similar results. C. T-DM1 and T-DXd significantly reduced tumor volume in HER2+ BC cell xenograft models. SUM190 or HCC1954 cells were injected into the mammary fat pad of nude mice, and the treatment was started when tumors averaged 200 mm^3^. HER2-ADC (10 mg/kg) was administered one time on Day 0, and tumor size was monitored. An IHC assay was used to check the expression levels of HER2 and proliferation maker Ki-67 in tumor samples. The data shown represent three IHC staining experiments from each treatment group with similar results. 20× magnification. Scale bars, 200 μm. *In vivo* tumorigenicity data were compared using an analysis of the variance model. ** *P* < 0.01, *** *P*< 0.001.   **Supplementary Figure S2.** A. HER2-ADC cell lines did not show an increase in genomic instability. Thirty-five metaphases/anaphases were analyzed per cell line. B. Deletion of the amplified ERBB2 region was observed on chromosome 17 in the SUM190-TDXdR cell line. Karyotyping assay. C. The intrinsic T-DXd-resistant KPL4 cell line (KPL4-TDXdR) did not show reduced ERBB2, MIEN1, MIR4728, and PGAP3 gene copy numbers. Whole-genome sequencing analysis. **Supplementary Figure S3.** A. Overexpression of HER2 did not increase the antiproliferation effect of T-DXd in T-DXd resistant HER2+ BC cell lines. SUM190-TDXdR and HCC1954-TDXdR cells transfected with pcDNA3-HER2 plasmid (Addgene, Watertown, MA, USA) using the Neon transfection kit (ThermoFisher) and underwent an SRB proliferation assay with T-DXd. The remaining cells were used for immunoblotting to check overexpression of HER2. B. Cells were transfected with validated siRNA targeting EGR1 or SLC6A14 (Silencer select Hm genome siRNA library v4, ThermoFisher) using the Neon transfection kit and incubated for 48 hr; they then underwent an SRB proliferation assay with T-DXd. Supplementary Figure S2. A. HER2-ADC cell lines did not show an increase in genomic instability. Thirty-five metaphases/anaphases were analyzed per cell line. B. Deletion of the amplified ERBB2 region was observed on chromosome 17 in the SUM190-TDXdR cell line. Karyotyping assay. C. The intrinsic T-DXd-resistant KPL4 cell line (KPL4-TDXdR) did not show reduced *ERBB2, MIEN1, MIR4728,* and *PGAP3* gene copy numbers. Whole-genome sequencing analysis. Supplementary Figure S3. A. Overexpression of HER2 did not increase the antiproliferation effect of T-DXd in T-DXd resistant HER2+ BC cell lines. SUM190-TDXdR and HCC1954-TDXdR cells transfected with pcDNA3-HER2 plasmid (Addgene, Watertown, MA, USA) using the Neon transfection kit (ThermoFisher) and underwent an SRB proliferation assay with T-DXd. The remaining cells were used for immunoblotting to check overexpression of HER2. B. Cells were transfected with validated siRNA targeting EGR1 or SLC6A14 (Silencer select Hm genome siRNA library v4, ThermoFisher) using the Neon transfection kit and incubated for 48 hr; they then underwent an SRB proliferation assay with T-DXd. **Supplementary Figure S4.** A. An Affymetrix Clariom D Human Transcriptome array data analysis identified targetable canonical pathways. TAC software was used to analyze and visualize global expression patterns of genes and pathways. The cut-off range was two-fold expression change (up and down) and *P* < 0.001. Significance was calculated using a 2x2 contingency in a Fisher’s exact test (two-sided). After the *P *value was established using Fisher’s exact test, it was converted to -log10. B-E. DNA repair pathway network analysis of microarray data from HER2-ADC-resistant cell lines. The cut-off range is the two-fold expression change (up and down) and *P* < 0.001. Significance was calculated using a 2x2 contingency in a Fisher’s Exact Test (two-sided). SUM190-TDM1R (B), HCC1954-TDM1R (C), SUM190-TDXdR (D), and HCC-1954TDXdR (E). Data were collected from three biological replicates. E-G. DNA repair pathway related proteins were elevated in HER2-ADC-resistant cell lines compared to is of microarray data from HER2-ADC-resistant cell lines. ATR, pATR, Chk1, Chk2, ATM, Rad50, and Rad51 were elevated in HER2-ADC-resistant cell lines. Reverse-phase protein array data (F). Hierarchical Clustering of reverse-phase protein array data using Morpheus software (https://software.broadinstitute.org/morpheus) Median expression values used for analysis (G). STRING interactome analysis of ATR, pATR, Chk1, Chk2, ATM, Rad50, and Rad51 (H). **Supplementary Figure S5.** DNA repair pathway–targeting drug enhances the efficacy of T-DXd in HER2-ADC-resistant HER2+ BC cell lines. A. Knockdown of ATR significantly reduces viability of HCC1954-TDXdR cell line. Two ATR RNAi were individually transfected, and SRB proliferation assay conducted for 7 days. B. ATR RNAi significantly enhances the efficacy of T-DXd in HER2+ BC. Cell lysates were collected at 72 hr after transfection for Western blotting analysis. The data shown are representative of three independent experiments with similar results. C and D. Clonogenic assay. Cells were treated with T-DXd and/or selected kinase inhibitor for 14 days, and cell viability was measured by SRB staining. SUM190-TDM1R and SUM190-TDXdR cell lines (C). HCC1954-TDM1R and HCC1954-TDXdR cell lines (D). Data are presented as mean ± standard deviation. Two-tailed unpaired Student’s *t*-test; *, *P* < 0.05, **, *P* < 0.01, ***, *P* < 0.001, ****, *P* < 0.0001, n.s. not significant. Experiments were repeated in triplicate. The data shown are representative of three independent experiments with similar results. E - H. Bliss independence dose-response assay. Cells were treated with T-DXd and selected inhibitors for 7 days, and viability was measured using SRB staining. The cell images were captured using GelCounter , the table indicates viability, and the Bliss synergy score was evaluated and visualized using the Synergyfinderplus software (right, www.synergyfinderplus.org). The color indicates a synergist (red) or antagonist (green) effect in two-drug combinations. **Supplementary Figure S6.** A. T-DXd and elimusertib increased DNA damage stress and apoptosis. Western blotting assay. Cells were treated with T-DXd (1 µg/ml) and/or elimusertib (100 nM) for 48 h, and whole-cell lysates were collected for immunoblotting. Protein expression was normalized with actin level in control cells from each TDM1R and TDXdR cell line using ImageJ software. B. T-DM1 or T-DXd treatment did not induce the HER2 downstream molecules, pAKT and pMAPK. Reverse-phase protein array data. Cells  C and D. Western blotting. Basal levels of HER, pHER2, pAKT, and pERK in parent and HER2-ADC-resistant cell lines (C). T-DXd treatment does not induces pAKT and pERK expression (D). The ImageJ program was used to measure intensity. **Supplementary Figure S7.** T-DXd and elimusertib did not show toxicity in xenograft models. T-DXd (10 mg/kg) was administered one time on Day 0 via tail-vein injection. Elimusertib (10 mg/kg) was administered via oral gavage twice a day (6-hr interval) for 3 consecutively days per week. Data are presented as mean ± standard deviation. **Supplementary Figure S8.** Reduced HER2 expression level is retained in HER2-ADC-resistant cell lines without T-DM1 or T-DMd treatment. To match xenograft assay conditions, HER2-ADC-resistant cell lines were maintained without the drug for 2 months. A. Western blotting. TDM1R and TDXdR cell lines retained reduced HER2 expression compared to the parent cell line. The ImageJ program was used for measuring intensity. B. FACS analysis. TDM1R and TDXdR cell lines showed reduced cell-surface HER2 expression. C. The sections (5-μm thick) were used for IHC staining, as described in the Methods section. The slides were then incubated with anti-HER2. Immunostained slides were scanned using an Aperio AT2 slide scanner and captured at 20× magnification using Aperio ImageScope software (Leica Biosystems). Scale bars = 200 µm. **Supplementary Figure S9.** Genes mutation profiling of the DNA repair pathway in HER2-ADC-resistant cell lines. Whole-genome sequencing data.

## Data Availability

The datasets generated and/or analyzed during the current study, including whole genome sequencing and microarray data, are available from the corresponding author upon request.

## Abbreviations

BC: Breast cancer
HER2+: Human epidermal growth factor receptor 2 positive
T-DM1: Trastuzumab emtansine
T-DXd: Trastuzumab deruxtecan
ADC: Antibody–drug conjugate
T-DM1R: T-DM1–resistant
T-DXdR: T-DXd–resistant
RNAi: RNA interference
IHC: Immunohistochemistry
TGI: Tumor growth inhibition
IRB: Institutional Review Board
GSEA: Gene Set Enrichment Analysis
FDR: False Discovery Rate
NES: Normalized Enrichment Score
CNV: Copy number variation
FFPE: Formalin-Fixed Paraffin-Embedded
FACS: Fluorescence-activated Cell Sorting
ATM: Ataxia Telangiectasia Mutated
ATR: Ataxia Telangiectasia and Rad3-Related
PARP: Poly (ADP-Ribose) Polymerase
STRING: Search Tool for the Retrieval of Interacting Genes/Proteins
