# Supplementary material for: The DNA repair pathway as a therapeutic target to synergize with trastuzumab deruxtecan in HER2-targeted antibody–drug conjugate–resistant HER2-overexpressing breast cancer
Source: J Exp Clin Cancer Res. 2024 Aug 21;43:236. doi: 10.1186/s13046-024-03143-3 (PMC11337831; doi:10.1186/s13046-024-03143-3)
Supplement: Supplementary file 2 — Supplementary Material 2: Supplementary Figure S1. Antiproliferation effect of T-DM1 and T-DXd in HER2-positive BC cell lines. A. HER2 gene copy variation in HER2+ BC cell lines. The TNBC cell line MDA-MB-231 was used as a negative control. Genomic DNA was used for the ddPCR assay. Each box shows mean with standard deviation. Data were collected from three biological replicates. B. T-DM1 and T-DXd inhibited the proliferation of HER2+ cell lines in a dose-dependent manner. Cells were treated with T-DM1 or T-DXd for 5 days, and viability was measured using SRB staining. The data shown are representative of 3 independent experiments with similar results. C. T-DM1 and T-DXd significantly reduced tumor volume in HER2+ BC cell xenograft models. SUM190 or HCC1954 cells were injected into the mammary fat pad of nude mice, and the treatment was started when tumors averaged 200 mm3. HER2-ADC (10 mg/kg) was administered one time on Day 0, and tumor size was monitored. An IHC assay was used to check the expression levels of HER2 and proliferation maker Ki-67 in tumor samples. The data shown represent three IHC staining experiments from each treatment group with similar results. 20× magnification. Scale bars, 200 μm. In vivo tumorigenicity data were compared using an analysis of the variance model. ** P < 0.01, *** P< 0.001. Supplementary Figure S2. A. HER2-ADC cell lines did not show an increase in genomic instability. Thirty-five metaphases/anaphases were analyzed per cell line. B. Deletion of the amplified ERBB2 region was observed on chromosome 17 in the SUM190-TDXdR cell line. Karyotyping assay. C. The intrinsic T-DXd-resistant KPL4 cell line (KPL4-TDXdR) did not show reduced ERBB2, MIEN1, MIR4728, and PGAP3 gene copy numbers. Whole-genome sequencing analysis. Supplementary Figure S3. A. Overexpression of HER2 did not increase the antiproliferation effect of T-DXd in T-DXd resistant HER2+ BC cell lines. SUM190-TDXdR and HCC1954-TDXdR cells transfected with pcDNA3-HER2 plasm [file 13046_2024_3143_MOESM2_ESM.pdf]

SUPPLEMENTARY MATERIALS

Targeting DNA repair pathway to synergize with trastuzumab deruxtecan in HER2-targeted antibody-drug conjugate-resistant HER2-overexpressing breast cancer

Jangsoon Lee, Kumiko Kida, Jiwon Koh, Huey Liu, Ganiraju C. Manyam, Young Jin Gi, Dileep R. Rampa, Asha S. Multani, Jing Wang, Gitanjali Jayachandran, Dae-Won Lee, James M. Reuben, Aysegul Sahin , Lei Huo, Debu Tripathy, Seock-Ah Im, and Naoto T. Ueno

Materials and Methods

Sulforhodamine B cell proliferation assay

The anti-proliferation effects of HER2-ADC and selected inhibitors against HER2+ cells were assessed using sulforhodamine B staining assays [1]. In brief, 3 to 10 × 103 cells/well were seeded in 96-well plates for short-term treatment (5 days) or 1 to 3 × 103 cells/well were seeded in 12-well plates for long-term treatment (14 days). The next day, cells were treated with HER2-ADC, alone or in combination with kinase inhibitors. The cells were then fixed with 5% trichloroacetic acid (Sigma-Aldrich) for 2 h at 4 ℃ and then stained with 0.03% sulforhodamine B solution (Sigma-Aldrich) for 30 min at room temperature. After being washed with 1% acetic acid three times, the stained cells were dissolved in 10 mM Tris buffer (Bio-Rad, Hercules, CA, USA). Optical density was determined at 480 (Ex)/590 (Em) nm using the VICTOR X3 plate reader (PerkinElmer, Waltham, MA, USA) or Spark microplate reader (Tecan, Männedorf Switzerland). The combination effect of T-DXd and elimusertib was determined using a Bliss independence dose-response surface model and visualized via the SynergyFinder Plus web application (www.synergyfinderplus.org) [2, 3].

High-throughput RNAi screening

Non-biased kinome library RNAi screening was performed as described in previous studies [4, 5]. In brief, three unique siRNAs targeting one gene and a total of 2127 siRNAs targeting 709 kinase genes were selected from Ambion Silencer Select Human Genome siRNA Library V4 (Life Technologies) and added to Greiner Bio-One CELLSTAR 384-Well plates (Thermo Fisher Scientific, Waltham, MA, USA). Seventy-five microliters of three pooled siRNAs (2 µM) for one gene were dispensed per well in quadruplicate. For an internal control, Silencer Select Negative Control No. 1 siRNA (Thermo Fisher Scientific), Silencer Select Positive Control PLK1 siRNA (Thermo Fisher Scientific), and no-siRNA control (Thermo Fisher Scientific) were included in each plate. The positive control PLK1 siRNA was used to assess transfection efficiency, and the no-siRNA control was used to calculate cellular sensitivity to T-DXd. Lipofectamine RNAiMAX (0.05 µL/well; Invitrogen) in 10 µL of serum-free Opti-MEM was added to the plates, and the plates were incubated at room temperature for 45 minutes. After incubation, SUM190-TDM1R or SUM190-TDXdR (300 cells) was added in 20 µL of complete media without antibiotics. The plates were sealed and incubated at 37 °C with 5% CO_2 for 48 h. A total of 28 plates were prepared for kinome screening, containing pooled siRNAs targeting 26 genes. At 48 h after treatment with pooled siRNAs, T-DXd at the final concentration of IC_{20} (0.3 nM) or vehicle (DMSO) in 30 µL of complete media was added to each well. The plates were then sealed and incubated at 37 °C with 5% CO_2 for 72 h. Following drug treatment, 35 µL of media was aspirated from each well, followed by the addition of ATPlite 1step (25 µL/well; PerkinElmer, Waltham, MA, USA). The plates were sealed and incubated at room temperature for 10 min, shaking at 1000 rpm on an orbital shaker. Luminescence readings were used as an indicator of cell viability. Luminescence readings for each treatment were averaged and normalized to the mean of the DMSO-treated no-siRNA negative control in the same plate to determine relative viability. PLK1-positive control and no-siRNA control were used to calculate each plate's z´-factor ($\boldsymbol{Z'} factor = \frac{\mathbf{3} \times (\boldsymbol{\sigma}\mathbf{p} + \boldsymbol{\sigma}\mathbf{n})}{|\boldsymbol{\mu}\mathbf{p} - \boldsymbol{\mu}\mathbf{n}|}$, where σp is the standard deviation of the

positive control, σn is the standard deviation of the negative control, μp is the mean of the positive control, and μn is the mean of the negative control).

To prioritize potential target whose inhibition could enhance the efficacy of T-DXd in HER-ADC-resistant BC cell liens, we assessed the sensitivity index. The median-centered normalization was applied across all siRNAs using the no-siRNA control on each plate to minimize plate-to-plate variability. The sensitivity index score, which assesses the impact of a specific siRNA-induced gene knockdown on T-DXd sensitivity, was evaluated for each siRNA following T-DXd treatment. This calculation was performed for every experiment and aligns with the methods described previously [6, 7]. The sensitivity index scores range from -1 to +1, with positive values indicating sensitizing effect of the T-DXd. The sensitivity index values ≥ 0.14 and a Z-score > 3 were considered as potential targets for combination with T-DXd.

Fluorescence *in situ* hybridization (FISH) analysis

Fluorescence *in situ* (FISH) assays were performed on the above cytological preparations using Her2 FISH probe and chromosome 17 centromeric probe (Empire Genomics Buffalo, NY, USA). The slides were hybridized with the FISH probes according to the manufacturer's instructions with slight modifications. Briefly, 2 μL of each of the two probes were mixed with 6 μl of the *in situ* hybridization buffer and applied to the slide, covered with a glass coverslip (22 × 22 mm), and sealed with rubber cement. The slides were then denatured at 72-73 °C using the ThermoBrite system (Abbott Laboratories, Abbott Park, IL, USA) and incubated at 37 °C overnight. They were then washed using 2× saline-sodium citrate 45-70 °C for 1-2 min and counterstained with DAPI. The analysis and imaging were performed using a Nikon Eclipse 80i microscope equipped with multiple filters.

Microarray analysis

Total RNA was extracted from parent and HER2-ADC resistant cell lines using the TRIzol Reagent and Phasemaker Tubes (Thermo Fisher Scientific) and submitted to the Advanced Technology Genomics Core for microarray analysis using Affymetrix Clariom D Human Transcriptome arrays (Thermo Fisher Scientific). Transcriptome Analysis Console Software (Thermo Fisher Scientific, (https://www.thermofisher.com/us/en/home/life-science/microarray-analysis/microarray-analysis-instruments-software-services/microarray-analysis-software/affymetrix-transcriptome-analysis-console-software.html)) was used to perform array quality control and data normalization, perform statistical tests for the differential expression of genes or pathways of interest, interpret complex alternative splicing events, and obtain sequence information to design validation experiments. Significance of gene expression is automatically calculated by TAC software using a 2x2 contingency table of the four elements (A-D, described below) with Fisher's Exact Test (Two Sided. **A** = Number of overlapping genes in this pathway (genes that passed the filtering criteria for a given pathway), **B** = Number of non-overlapping genes in this pathway (genes that do not pass the filtering criteria for a given pathway), **C** = Number of genes in current table that passed the filtering criteria but do not belong to this pathway. **D** = (Total number of genes that did not pass the filtering criteria) – B After a *p*-value is established using Fisher's Exact Test, it is converted to -log10. The result of this conversion is the Significance. Detailed analysis algorithm is described in manufactures instruction (https://assets.thermofisher.com/TFS-Assets/LSG/manuals/tac_user_manual.pdf)

We also performed an analysis using the oligo package of Statistical software R (version 4.1.3, https://www.r-project.org/). Normalization was performed using the RMA algorithm. A differential expression analysis was performed using a *t*-test between the contrasts of interest. The *P* values obtained by multiple *t*-tests were corrected by the Benjamini-Hochberg method.

Droplet digital PCR (ddPCR) assay

The ddPCR assay was performed using the QX200 system (Bio-Rad) according to the manufacturer's recommendations. In brief, DNA isolation was performed using the PureLink Genomic DNA Mini Kit (Thermo Fisher Scientific). The samples were processed for ddPCR in a blinded fashion. Restriction enzyme digestion of the genomic DNA with MSE I was performed prior to droplet generation. About 200 ng of DNA was used for restriction enzyme digestion in a final reaction volume of 10 µL. The amplification reaction included the digested genomic DNA (3 µL), 4× ddPCR Multiplex Supermix for probes (Bio-Rad) and 20× copy number variation assays in a final volume of 20 µL. The ddPCR copy number variation assays were wet-laboratory-validated in-house by the manufacturer and were used for both the target gene *HER2* (FAM) and for as the reference gene *RPP30* (HEX). The reaction mixtures were partitioned into an emulsion of approximately 20,000 droplets in oil by using 70 µL of droplet generation oil (Bio-Rad) and 20 µL of PCR reaction mix loaded into a disposable plastic cartridge (Bio-Rad) and placed in the droplet generator. After processing, the droplets obtained in a 40-µL volume from each sample were transferred to a 96-well PCR plate, and amplification was carried out using a T100 Thermal Cycler (Bio-Rad) with the following cycling conditions: DNA polymerase activation at 95 °C for 10 min followed by 40 cycles of PCR amplification (94 °C for 30 s and 60 °C for 60 s), and 98 °C for 10 min, 2 °C/s ramp rate at all steps. After PCR, the droplets were counted with the QX200 Droplet Reader using Bio-Rad QX Manager Standard Edition version 1.2 software. The *HER2*/*RPP30* copy number ratio was analyzed by calculating the copies per droplet from the Poisson distribution.

Small Interference RNA (siRNA) Selection and Transfection

Pre-validated siRNAs were selected from the Silencer Select Human Genome siRNA Library v4 (Thermo Fisher Scientific). For EGR1 (NM_001964), the sequences were: 5'-CAACGACAGCAGUCCCAUUtt-3' and 5'-AAUGGGACUGCUGUCGUUGga-3'; 5'-CCAUGGACAACUACCCUAAtt-3' and 5'-UUAGGGUAGUUGUCCAUGGtg-3'; 5'-

GGACAUGACAGCAACCUUUtt-3’ and 5’-AAAGGUUGCUGUCAUGUCCga-3’. For SLC6A14 (NM_007231), the sequences were: 5’-GGGACAAUUUGCUAGCUUAtt-3’ and 5’-UAAGCUAGCAAAUUGUCCCag-3’; 5’-GGGUUCAUCUGAUUGACCAtt-3’ and 5’-UGGUCAAUCAGAUGAACCCag-3’; 5’-CGGAUUAUCUUCUAUCUAUtt-3’ and 5’-AUAGAUAGAAGAUAAUCCGat-3’. For ATR (NM_001184), the sequences were: 5’-GAUCCUACAUCAUGGUACAtt-3’ and 5’- UGUACCAUGAUGUAGGAUCtt-3’; 5’-GACAUUGCAUCUGAUCUGUtt-3’ and 5’-ACAGAUCAGAUGCAAUGUCtt-3’.

Three siRNAs were pooled and transfected into cells using the Neon Transfection System with the 100-µL Kit (Life Technologies) according to the manufacturer's instructions. In brief, cells were harvested using 0.1× TrypLE Express (Thermo Fisher Scientific), counted, and resuspended in resuspension buffer R at a density of 0.5×10^7 cells/mL. An aliquot of 100 µL of cells was transferred into sample tubes, and 10 µL of the siRNA mixture was added to achieve a final 200 nM siRNA concentration. The transfection parameters were set at 1200V, 20 ms, 3 pulses for all cell lines. Cells were incubated for 36-48 hours, harvested, counted, and seeded into a 96-well plate to conduct the SRB proliferation assay.

Whole-genome sequencing analysis

Genomic DNA was extracted from parent and HER2-ADC resistant cell lines using the PureLink Genomic DNA Mini Kit (Thermo Fisher Scientific) and submitted to Azenta (Burlington, MA, USA) for next-generation sequencing analysis using the Illumina HiSeq 4000 with 90Gb coverage. In brief, genomic DNA was quantified using the Qubit 2.0 Fluorometer (Thermo Fisher Scientific). The NEBNext Ultra DNA Library Prep Kit for Illumina, clustering, and sequencing reagents was used throughout the process following the manufacturer’s recommendations. Briefly, 500 ng of the genomic DNA was fragmented by acoustic shearing with a Covaris S220 instrument. Fragmented DNA was cleaned up and end-repaired. Adapters were ligated after adenylation of the 3′ ends followed by

enrichment by limited-cycle PCR. DNA libraries were validated using a High Sensitivity D1000 ScreenTape on the Agilent TapeStation (Agilent Technologies, Palo Alto, CA, USA) and quantified using the Qubit 2.0 Fluorometer. The sequencing library was clustered onto a lane of an Illumina HiSeq 4000 flow cell. After clustering, the flow cell was loaded onto the Illumina HiSeq instrument, according to the manufacturer's instructions. The samples were sequenced using a 2 × 150 bp paired end configuration. Image analysis and base calling were conducted by the HiSeq Control Software. Raw sequence data (.bcl files) generated from Illumina HiSeq were converted into FASTQ files and de-multiplexed using Illumina bcl2fastq 2.17 software. One mismatch was allowed for index sequence identification.

Karyotyping by G-banding and genomic instability analysis

Cells were cultured in complete media supplemented with 10% fetal bovine serum, and chromosome preparations were made following the standard air-drying technique. Aged slides were G-banded, using 0.05% trypsin EDTA solution (Thermo Fisher Scientific). G-banded metaphase spreads were photographed using a 80i Nikon Microscope and Applied Spectral Imaging Karyotyping system. A minimum of 10 metaphases were karyotyped. For the genomic instability analysis, cytological preparations were stained in 4% Giemsa solution, and the slides were analyzed for chromosomal aberrations, including chromosome and chromatid breaks, fusions, fragments, and tetraploidy. A minimum of 35 metaphases/anaphases were analyzed per cell line.

Western blotting

Cells (3×10^5 cells/10 mL) were seeded in 6-cm plates overnight and the next day treated with T-DXd alone or in combination with elimusertib for 48 h. Total protein was extracted using M-PER Mammalian

Protein Extraction Reagent (Thermo Fisher Scientific) complemented with phosphatase and protease inhibitors (Bimake). The protein samples (15 μg) were denatured by incubation with NuPAGE LDS Sample Buffer (Thermo Fisher Scientific) and Sample Reducing Agent (Thermo Fisher Scientific) at 70 °C for 10 min, resolved using NuPAGE 4–12% Bis-Tris Plus gel (Thermo Fisher Scientific), and transferred onto a polyvinylidene difluoride membrane (Bio-Rad). Following blocking with 4% bovine serum albumin, proteins of interest on the blots were probed using the primary antibodies. The secondary antibodies used were horseradish peroxidase–conjugated immunoglobulin G (Thermo Fisher Scientific) for chemiluminescence signal detection (Thermo Fisher Scientific). The intensity of target proteins on the blots was captured using the ImageQuant LAS 4000 imager (Cytiva, Marlborough, MA, USA).

Supplementary Figure legends

Supplementary Figure S1. Antiproliferation effect of T-DM1 and T-DXd in HER2-positive BC cell lines.

A. HER2 gene copy variation in HER2+ BC cell lines. The TNBC cell line MDA-MB-231 was used as a negative control. Genomic DNA was used for the ddPCR assay. Each box shows mean with standard deviation. Data were collected from three biological replicates. **B.** T-DM1 and T-DXd inhibited the proliferation of HER2+ cell lines in a dose-dependent manner. Cells were treated with T-DM1 or T-DXd for 5 days, and viability was measured using SRB staining. The data shown are representative of 3 independent experiments with similar results. **C**. T-DM1 and T-DXd significantly reduced tumor volume in HER2+ BC cell xenograft models. SUM190 or HCC1954 cells were injected into the mammary fat pad of nude mice, and the treatment was started when tumors averaged 200 mm^3. HER2-ADC (10 mg/kg) was administered one time on Day 0, and tumor size was monitored. An IHC assay was used to check the expression levels of HER2 and proliferation maker Ki-67 in tumor samples. The

data shown represent three IHC staining experiments from each treatment group with similar results. 20× magnification. Scale bars, 200 µm. *In vivo* tumorigenicity data were compared using an analysis of the variance model. ** $P < 0.01$, *** $P < 0.001$.

Supplementary Figure S2. A. HER2-ADC cell lines did not show an increase in genomic instability. Thirty-five metaphases/anaphases were analyzed per cell line. **B**. Deletion of the amplified ERBB2 region was observed on chromosome 17 in the SUM190-TDXdR cell line. Karyotyping assay. **C**. The intrinsic T-DXd-resistant KPL4 cell line (KPL4-TDXdR) did not show reduced *ERBB2, MIEN1, MIR4728,* and *PGAP3* gene copy numbers. Whole-genome sequencing analysis.

Supplementary Figure S3. A. Overexpression of HER2 did not increase the antiproliferation effect of T-DXd in T-DXd resistant HER2+ BC cell lines. SUM190-TDXdR and HCC1954-TDXdR cells transfected with pcDNA3-HER2 plasmid (Addgene, Watertown, MA, USA) using the Neon transfection kit (ThermoFisher) and underwent an SRB proliferation assay with T-DXd. The remaining cells were used for immunoblotting to check overexpression of HER2. **B**. Cells were transfected with validated siRNA targeting EGR1 or SLC6A14 (Silencer select Hm genome siRNA library v4, ThermoFisher) using the Neon transfection kit and incubated for 48 hr; they then underwent an SRB proliferation assay with T-DXd.

Supplementary Figure S4. A. An Affymetrix Clariom D Human Transcriptome array data analysis identified targetable canonical pathways. TAC software was used to analyze and visualize global expression patterns of genes and pathways. The cut-off range was two-fold expression change (up and down) and $P < 0.001$. Significance was calculated using a 2x2 contingency in a Fisher's exact test (two-sided). After the *P* value was established using Fisher's exact test, it was converted to -log10. **B-E**. DNA

repair pathway network analysis of microarray data from HER2-ADC-resistant cell lines. The cut-off range is the two-fold expression change (up and down) and *P* < 0.001. Significance was calculated using a 2x2 contingency in a Fisher's Exact Test (two-sided). SUM190-TDM1R (B), HCC1954-TDM1R (C), SUM190-TDXdR (D), and HCC-1954TDXdR (E). Data were collected from three biological replicates. **E-G**. DNA repair pathway related proteins were elevated in HER2-ADC-resistant cell lines compared to is of microarray data from HER2-ADC-resistant cell lines. ATR, pATR, Chk1, Chk2, ATM, Rad50, and Rad51 were elevated in HER2-ADC-resistant cell lines. Reverse-phase protein array data (F). Hierarchical Clustering of reverse-phase protein array data using Morpheus software (https://software.broadinstitute.org/morpheus) Median expression values used for analysis (G). STRING interactome analysis of ATR, pATR, Chk1, Chk2, ATM, Rad50, and Rad51 (H).

Supplementary Figure S5. DNA repair pathway–targeting drug enhances the efficacy of T-DXd in HER2-ADC-resistant HER2+ BC cell lines. **A**. Knockdown of ATR significantly reduces viability of HCC1954-TDXdR cell line. Two ATR RNAi were individually transfected, and SRB proliferation assay conducted for 7 days. **B**. ATR RNAi significantly enhances the efficacy of T-DXd in HER2+ BC. Cell lysates were collected at 72 hr after transfection for Western blotting analysis. The data shown are representative of three independent experiments with similar results. **C** and **D**. Clonogenic assay. Cells were treated with T-DXd and/or selected kinase inhibitor for 14 days, and cell viability was measured by SRB staining. SUM190-TDM1R and SUM190-TDXdR cell lines (C). HCC1954-TDM1R and HCC1954-TDXdR cell lines (D). Data are presented as mean ± standard deviation. Two-tailed unpaired Student's *t*-test; *, *P* < 0.05, **, *P* < 0.01, ***, *P* < 0.001, ****, *P* < 0.0001, n.s. not significant. Experiments were repeated in triplicate**.** The data shown are representative of three independent experiments with similar results. **E - H**. Bliss independence dose-response assay. Cells were treated with T-DXd and selected inhibitors for 7 days, and viability was measured using SRB staining. The cell images were captured using GelCounter , the table indicates viability, and the Bliss synergy score was

evaluated and visualized using the Synergyfinderplus software (right, www.synergyfinderplus.org). The color indicates a synergist (red) or antagonist (green) effect in two-drug combinations.

Supplementary Figure S6. **A**. T-DXd and elimusertib increased DNA damage stress and apoptosis. Western blotting assay. Cells were treated with T-DXd (1 μg/ml) and/or elimusertib (100 nM) for 48 h, and whole-cell lysates were collected for immunoblotting. Protein expression was normalized with actin level in control cells from each TDM1R and TDXdR cell line using ImageJ software. **B**. T-DM1 or T-DXd treatment did not induce the HER2 downstream molecules, pAKT and pMAPK. Reverse-phase protein array data. Cells **C** and **D**. Western blotting. Basal levels of HER, pHER2, pAKT, and pERK in parent and HER2-ADC-resistant cell lines (C). T-DXd treatment does not induces pAKT and pERK expression (D). The ImageJ program was used to measure intensity.

Supplementary Figure S7. T-DXd and elimusertib did not show toxicity in xenograft models. T-DXd (10 mg/kg) was administered one time on Day 0 via tail-vein injection. Elimusertib (10 mg/kg) was administered via oral gavage twice a day (6-hr interval) for 3 consecutively days per week. Data are presented as mean ± standard deviation.

Supplementary Figure S8. Reduced HER2 expression level is retained in HER2-ADC-resistant cell lines without T-DM1 or T-DMd treatment. To match xenograft assay conditions, HER2-ADC-resistant cell lines were maintained without the drug for 2 months. **A**. Western blotting. TDM1R and TDXdR cell lines retained reduced HER2 expression compared to the parent cell line. The ImageJ program was used for measuring intensity. **B**. FACS analysis. TDM1R and TDXdR cell lines showed reduced cell-surface HER2 expression. **C**. The sections (5-µm thick) were used for IHC staining, as described in the Methods section. The slides were then incubated with anti-HER2. Immunostained slides were scanned using an

Aperio AT2 slide scanner and captured at 20× magnification using Aperio ImageScope software (Leica Biosystems). Scale bars = 200 µm.

Supplementary Figure S9. Genes mutation profiling of the DNA repair pathway in HER2-ADC-resistant cell lines. Whole-genome sequencing data.

References.

1. Vichai V, Kirtikara K: **Sulforhodamine B colorimetric assay for cytotoxicity screening**. *Nat Protoc* 2006, **1**(3):1112-1116.
2. Zheng S, Wang W, Aldahdooh J, Malyutina A, Shadbahr T, Tanoli Z, Pessia A, Tang J: **SynergyFinder Plus: Toward Better Interpretation and Annotation of Drug Combination Screening Datasets**. *Genomics Proteomics Bioinformatics* 2022, **20**(3):587-596.
3. Liu Q, Yin X, Languino LR, Altieri DC: **Evaluation of drug combination effect using a Bliss independence dose-response surface model**. *Stat Biopharm Res* 2018, **10**(2):112-122.
4. Lee J, Galloway R, Grandjean G, Jacob J, Humphries J, Bartholomeusz C, Goodstal S, Lim B, Bartholomeusz G, Ueno NT, Rao A: **Comprehensive Two- and Three-Dimensional RNAi Screening Identifies PI3K Inhibition as a Complement to MEK Inhibitor AS703026 for Combination Treatment of Triple-Negative Breast Cancer**. *J Cancer* 2015, **6**(12):1306-1319.
5. Lee J, Liu H, Pearson T, Iwase T, Fuson J, Lalani AS, Eli LD, Diala I, Tripathy D, Lim B, Ueno NT: **PI3K and MAPK Pathways as Targets for Combination with the Pan-HER Irreversible Inhibitor Neratinib in HER2-Positive Breast Cancer and TNBC by Kinome RNAi Screening**. *Biomedicines* 2021, **9**(7).
6. Jansen VM, Bhola NE, Bauer JA, Formisano L, Lee KM, Hutchinson KE, Witkiewicz AK, Moore PD, Estrada MV, Sanchez V *et al*: **Kinome-Wide RNA Interference Screen Reveals a

Role for PDK1 in Acquired Resistance to CDK4/6 Inhibition in ER-Positive Breast Cancer. *Cancer Res* 2017, **77**(9):2488-2499.

7. Bauer JA, Ye F, Marshall CB, Lehmann BD, Pendleton CS, Shyr Y, Arteaga CL, Pietenpol JA: **RNA interference (RNAi) screening approach identifies agents that enhance paclitaxel activity in breast cancer cells**. *Breast Cancer Res* 2010, **12**(3):R41.

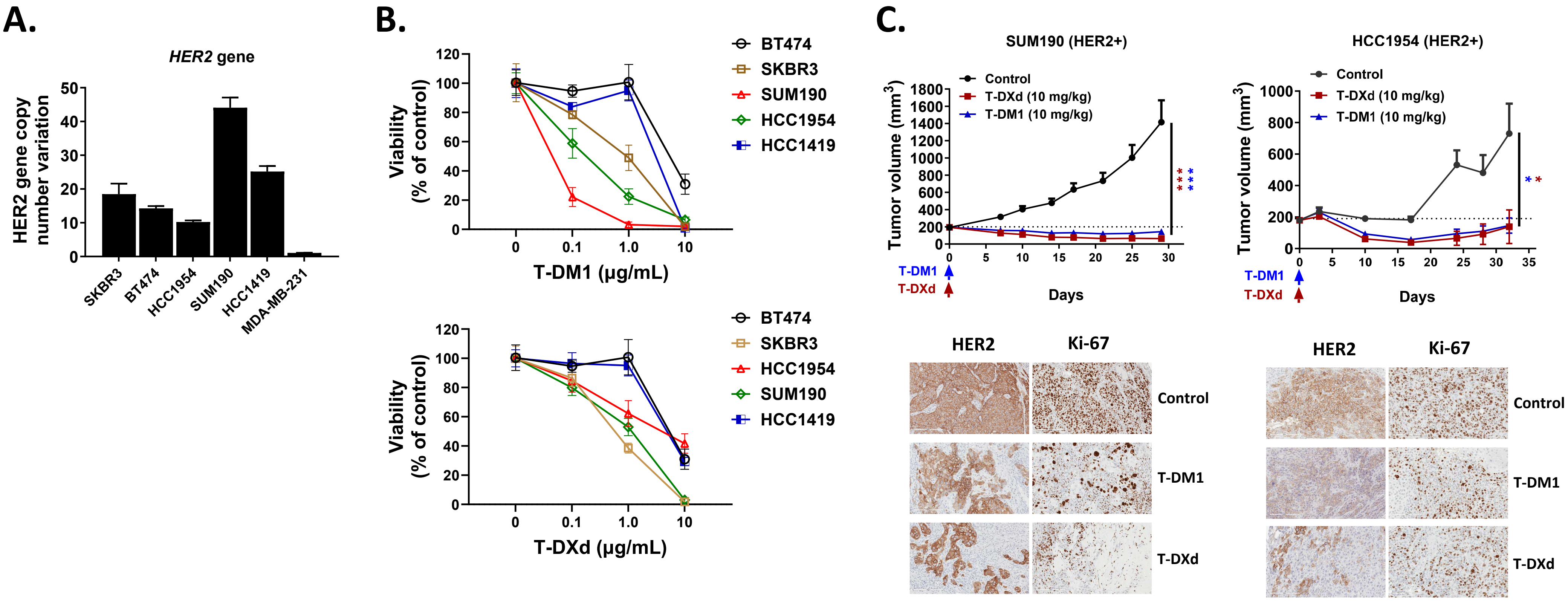

Supplemental Fig. S1

A.

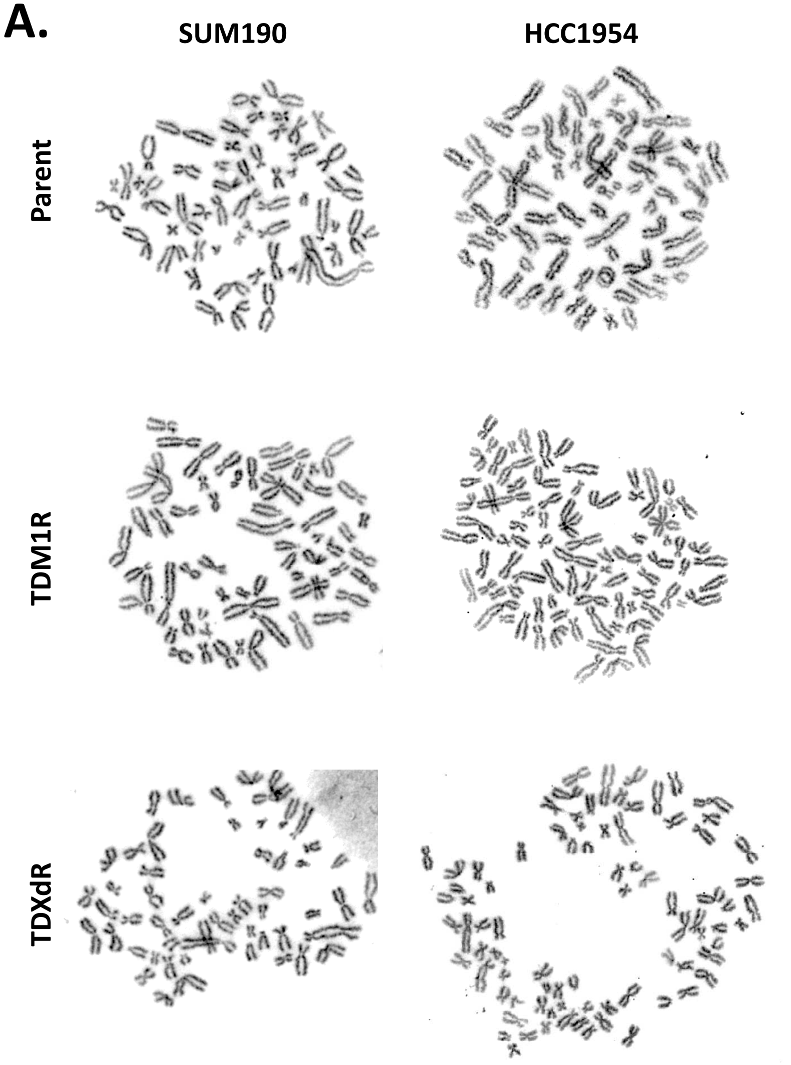

Sample	No. of meta. analysis	no. of normal looking meta	no. of aberr. meta	no. of cells with breaks	No. of cells with fusions	No. of tetra/polyploid cells	No. of cells with C-anaphase morphology	Comment
SUM190	35	34	1	1	0	0	0	
SUM190-TDM1R	35	34	1	0	1	0	0	
SUM190-TDXdR	35	34	1	0	1	0	0	
HCC1954	35	31	4	2	3	1	0	1 cell with many aberrations
HCC1954-TDM1R	35	32	3	2	2	0	0	
HCC1954-TDXdR	35	32	3	1	2	0	0	

Supplemental Fig. S2

B.

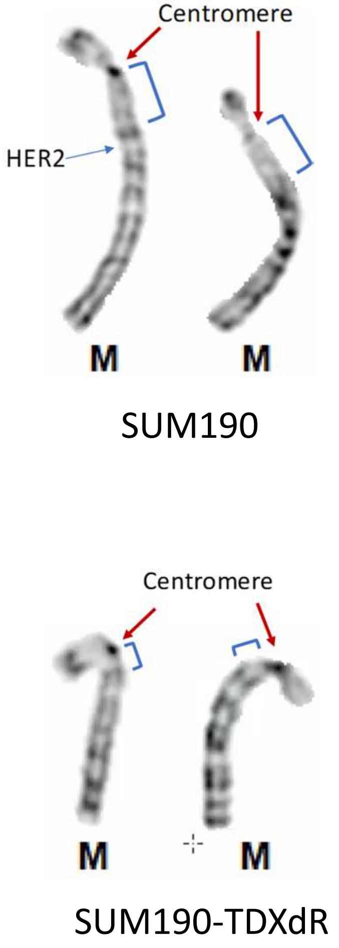

C.

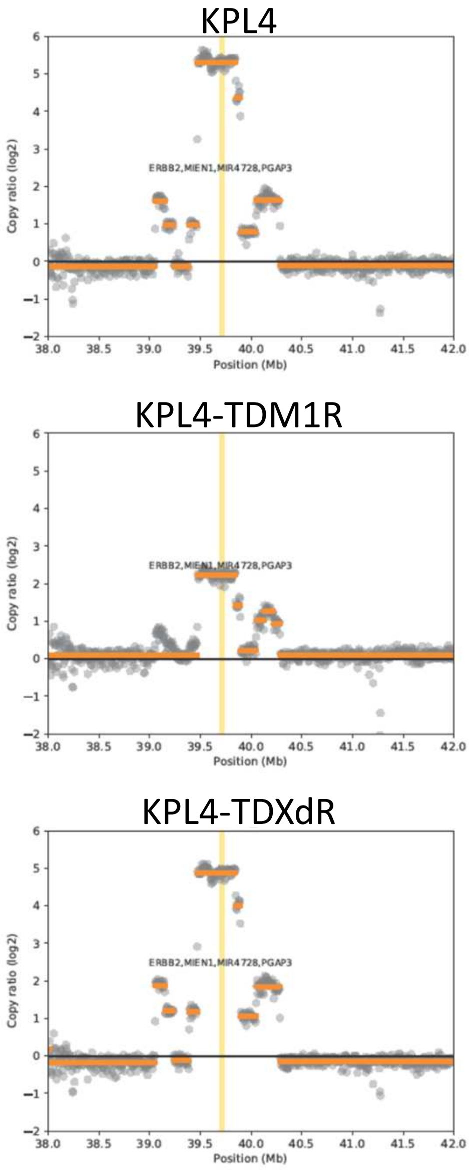

Supplemental Fig. S2

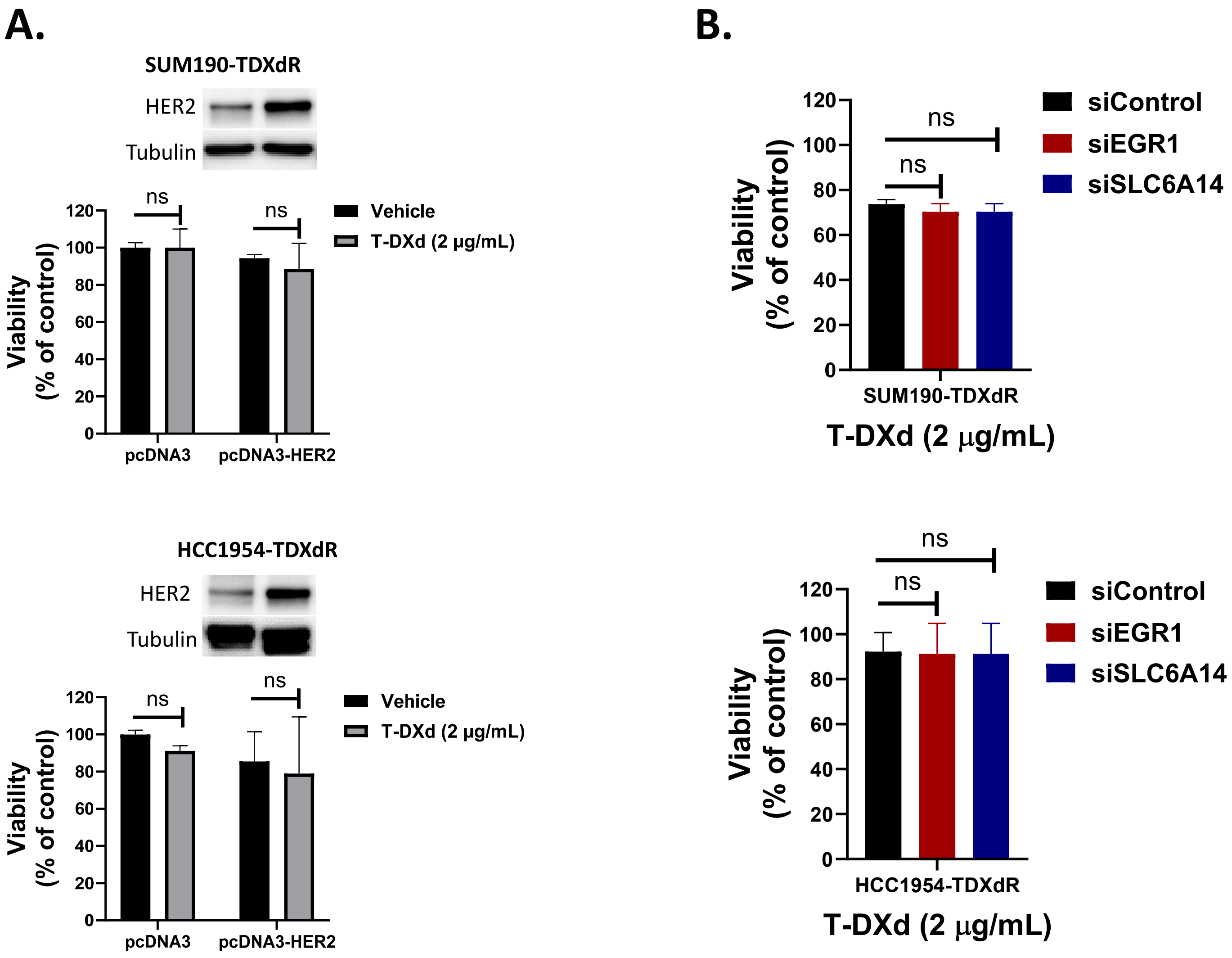

Supplemental Fig. S3

A.

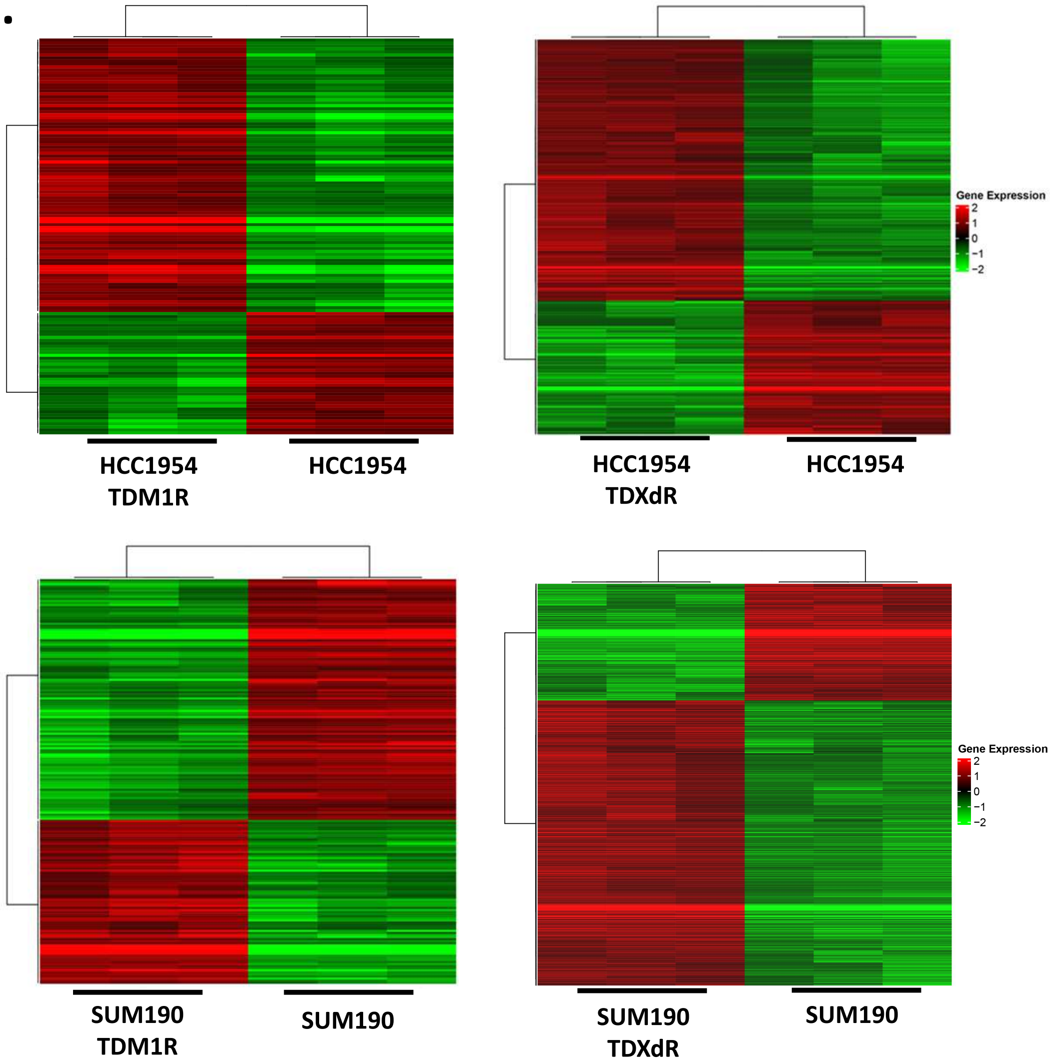

Activated canonical pathways in HER2-ADC-resistant HER2+ BC cell lines

Pathways	Significance (*P*-value)			
	SUM190 TDM1R	SUM190 TDXdR	HCC1954 TDM1R	HCC1954 TDXdR
AXL signaling	2.5E-07	3.1E-08	3.1E-08	2.0E-05
DNA damage response	6.3E-05	5.6E-11	1.7E-04	1.5E-04
EGF/EGFR signaling	2.2E-07	6.2E-11	1.3E-08	3.7E-06
Focal adhesion	4.4E-07	9.1E-08	9.5E-10	1.7E-04
Glucocorticoid receptor	2.3E-10	4.8E-12	9.5E-10	3.0E-05
Nuclear receptors meta	4.7E-21	3.1E-22	3.1E-13	9.8E-10
PI3K-Akt signaling	1.8E-14	2.2E-12	2.3E-07	1.5E-05
TGF-beta signaling	8.7E-09	4.6E-19	2.5E-06	6.8E-06
VEGFA-VEGFR2 signaling	1.3E-15	8.1E-27	5.2E-11	3.8E-11

Analyzed by Transcriptome Analysis Console (V.4.0.2). Fold change, >2 or <2; FDR *p*-value <0.01

Supplemental Fig. S4

B.

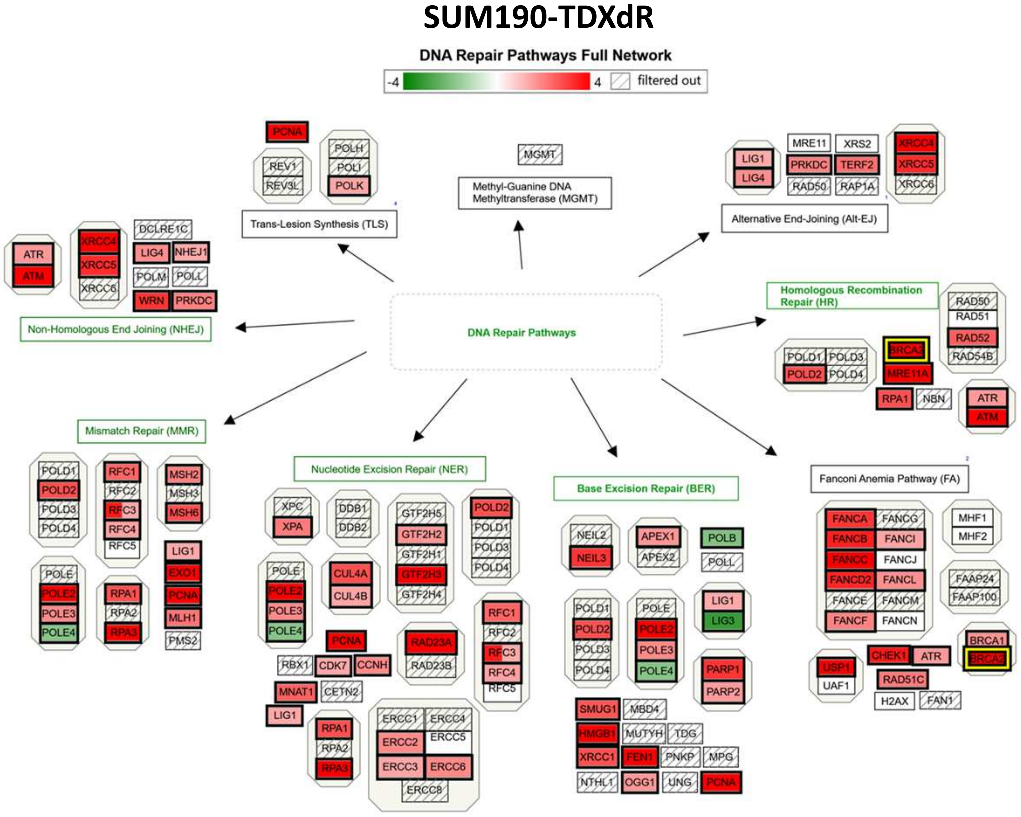

C.

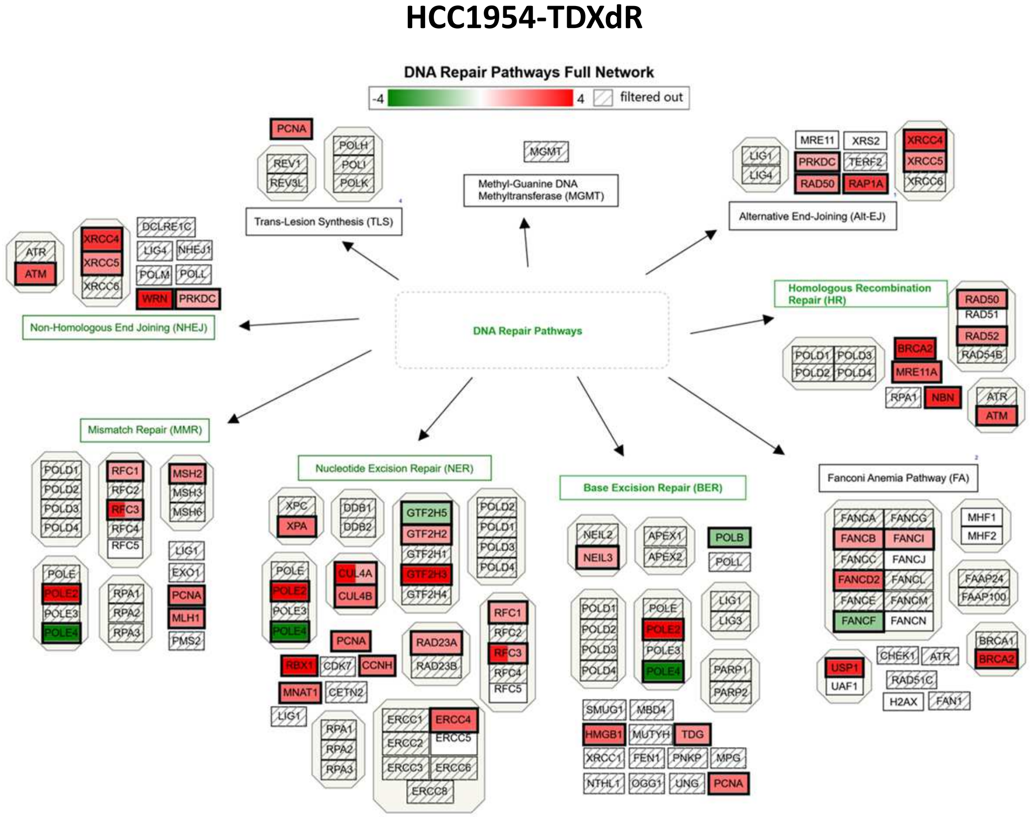

Supplemental Fig. S4

D.

SUM190-TDM1R

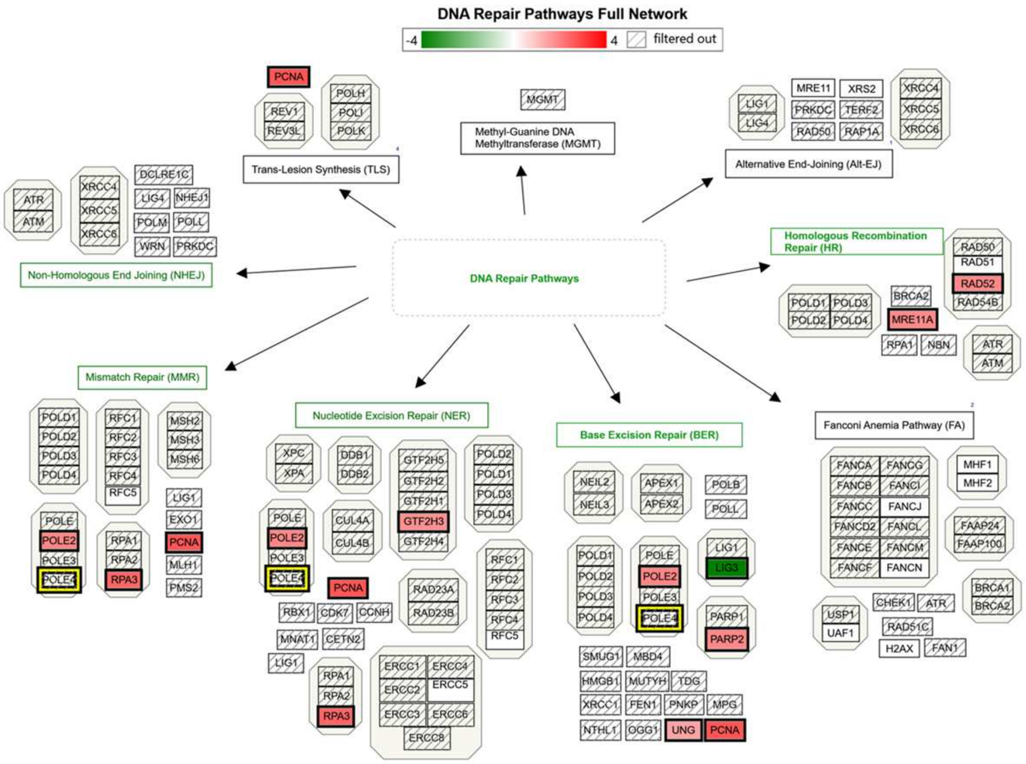

E.

HCC1954-TDM1R

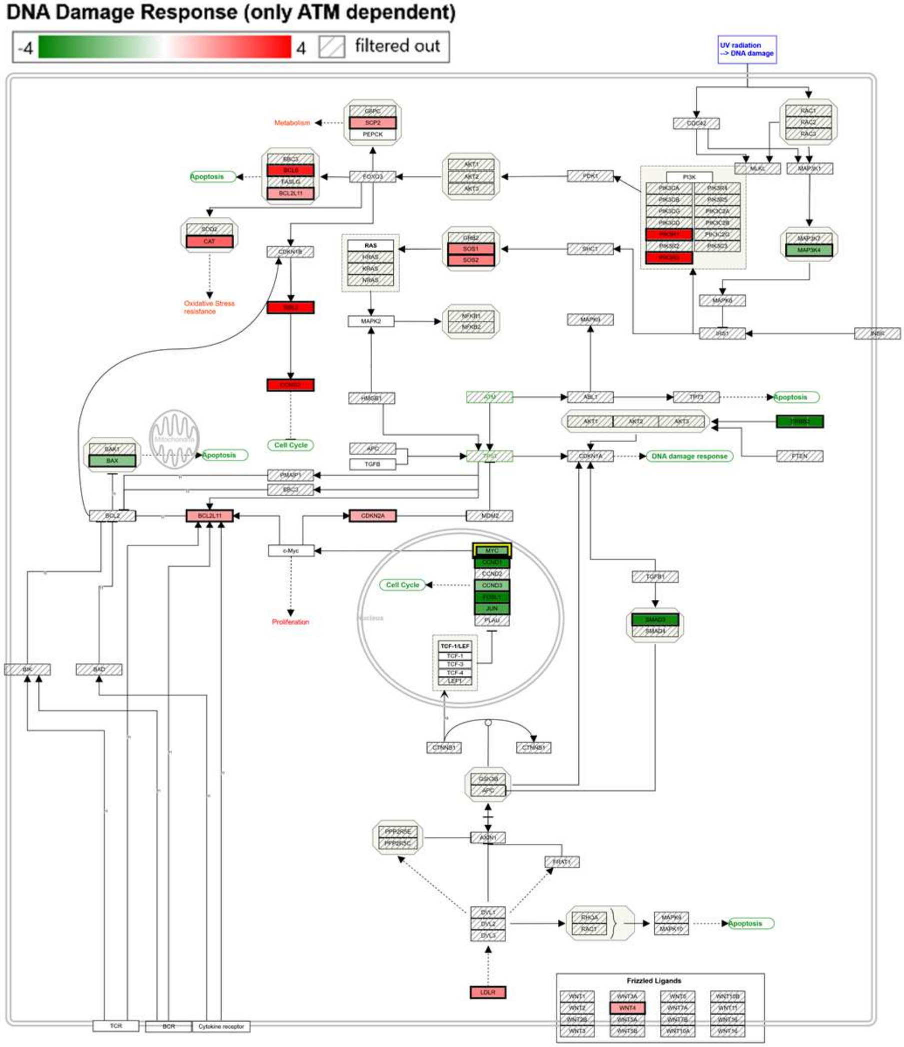

Supplemental Fig. S4

F.

	ATM	ATR	ATR_pS428	Chk1	Chk2	Rad50	Rad51
**SUM190**	-0.133	0.070	-0.188	-0.058	0.000	-0.329	-0.095
**SUM190**	-0.123	-0.014	-0.184	-0.140	-0.214	-0.294	0.009
**SUM190**	-0.323	0.059	-0.358	-0.015	-0.008	-0.413	-0.034
**SUM190-TDM1R**	0.000	-0.042	-0.060	-0.004	-0.122	0.035	0.068
**SUM190-TDM1R**	0.081	-0.078	0.065	-0.026	-0.167	0.111	-0.057
**SUM190-TDM1R**	-0.149	-0.006	-0.167	-0.059	-0.026	-0.014	-0.124
**SUM190-TDXdR**	0.348	-0.072	0.098	0.163	0.092	0.261	0.009
**SUM190-TDXdR**	0.392	0.142	0.190	0.231	0.267	0.331	0.293
**SUM190-TDXdR**	0.369	0.148	0.049	0.307	0.209	0.296	0.396

	ATM	ATR	ATR_pS428	Chk1	Chk2	Rad50	Rad51
**HCC1954**	-0.161	0.115	-0.202	-0.090	0.091	-0.188	0.041
**HCC1954**	-0.024	-0.002	-0.011	-0.076	0.017	-0.016	0.033
**HCC1954**	-0.059	0.050	-0.105	-0.112	0.038	-0.084	-0.035
**HCC1954-TDM1R**	0.010	0.000	0.096	0.173	-0.158	-0.057	-0.061
**HCC1954-TDM1R**	-0.106	-0.006	0.075	0.074	-0.177	-0.078	0.073
**HCC1954-TDM1R**	-0.107	0.026	0.060	0.035	-0.141	-0.081	-0.083
**HCC1954-TDXdR**	0.118	-0.095	0.024	0.003	0.189	0.145	0.113
**HCC1954-TDXdR**	0.267	-0.019	-0.029	0.014	0.239	0.109	0.149
**HCC1954-TDXdR**	0.262	-0.077	0.058	-0.009	0.211	0.249	0.174

G.

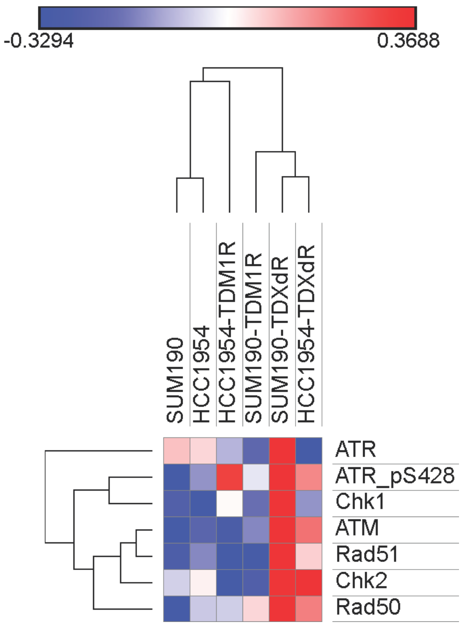

H.

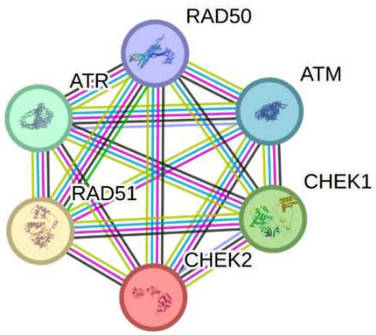

Supplemental Fig. S4

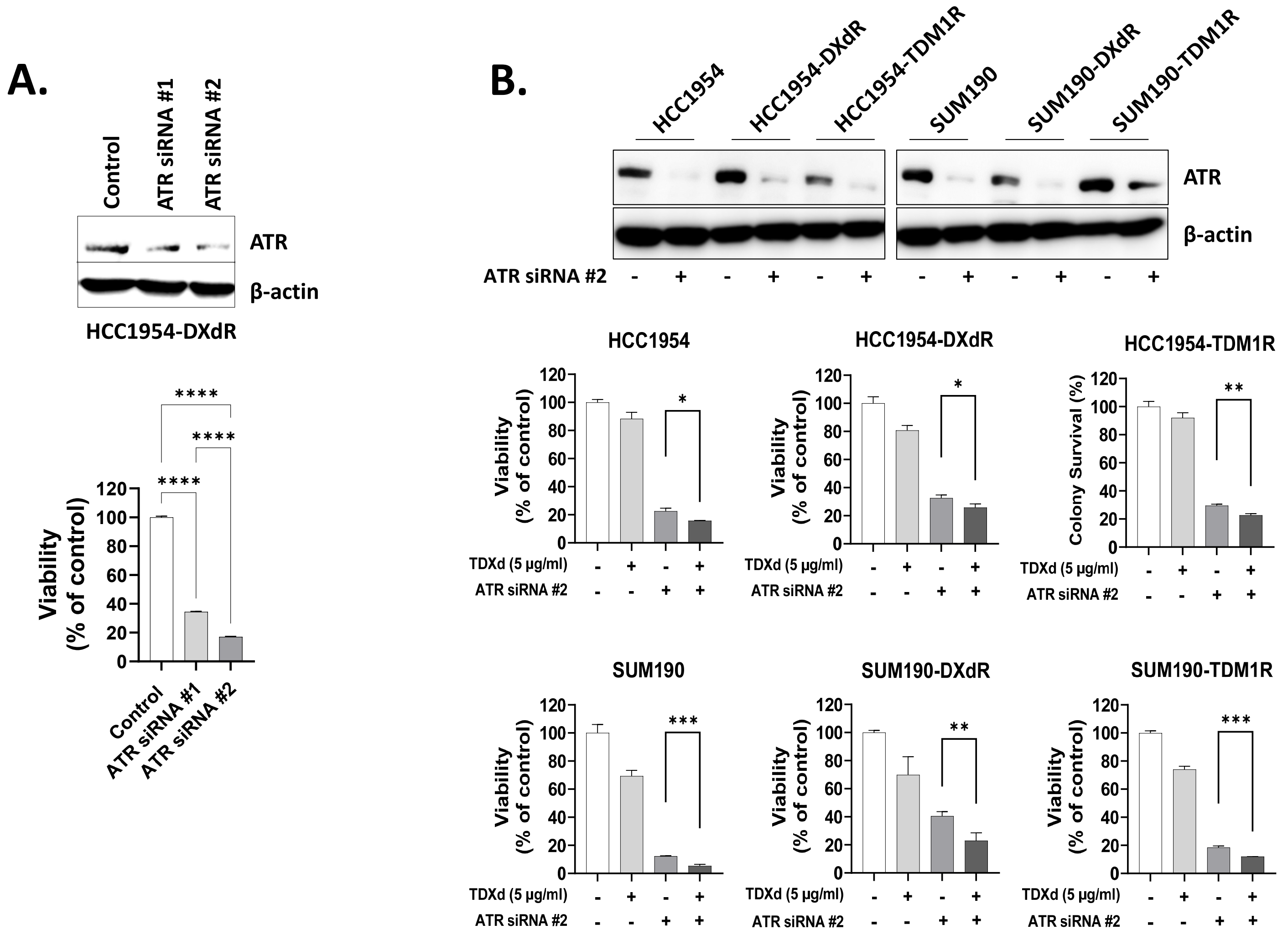

Supplemental Fig. S5

C.

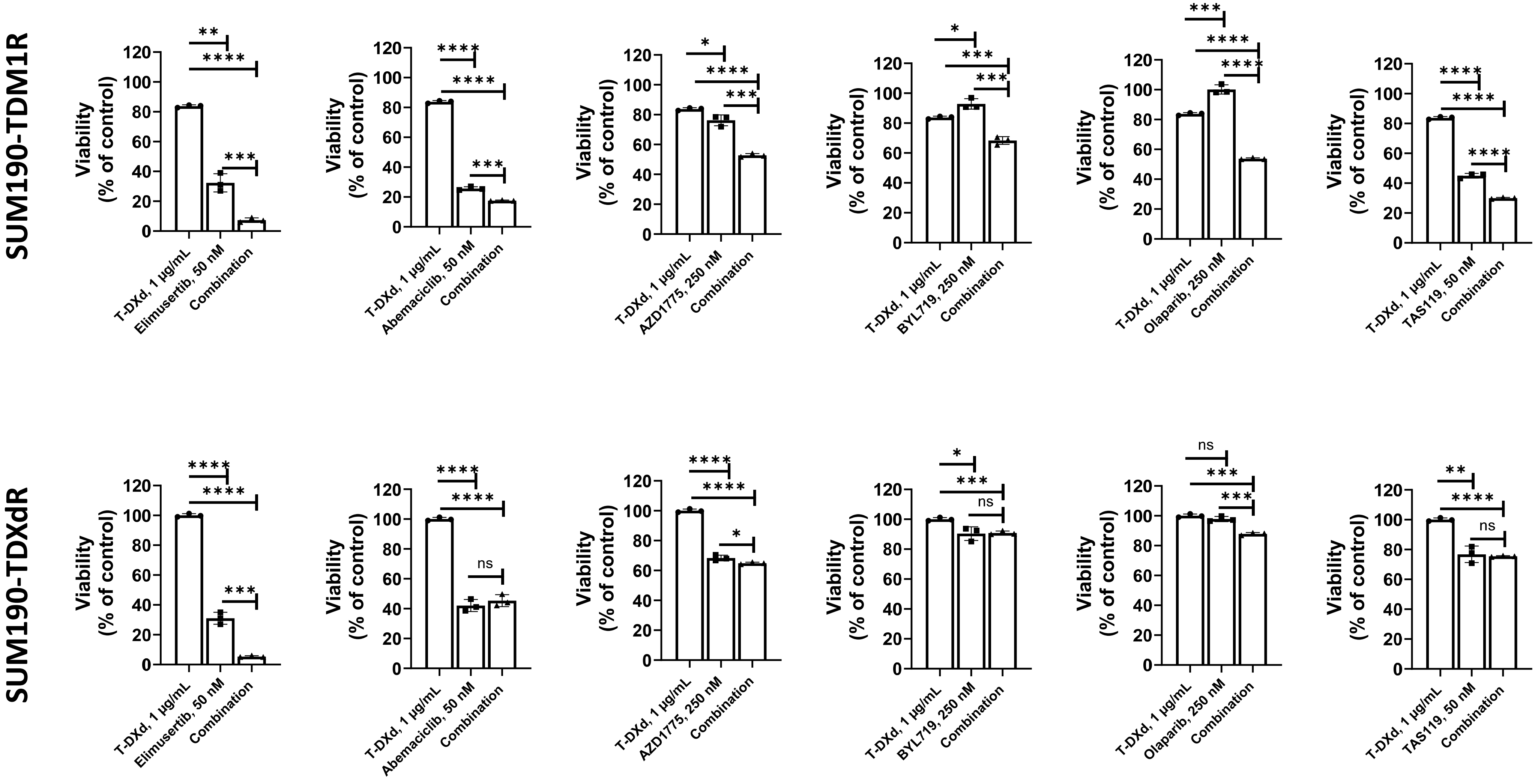

Supplemental Fig. S5

D.

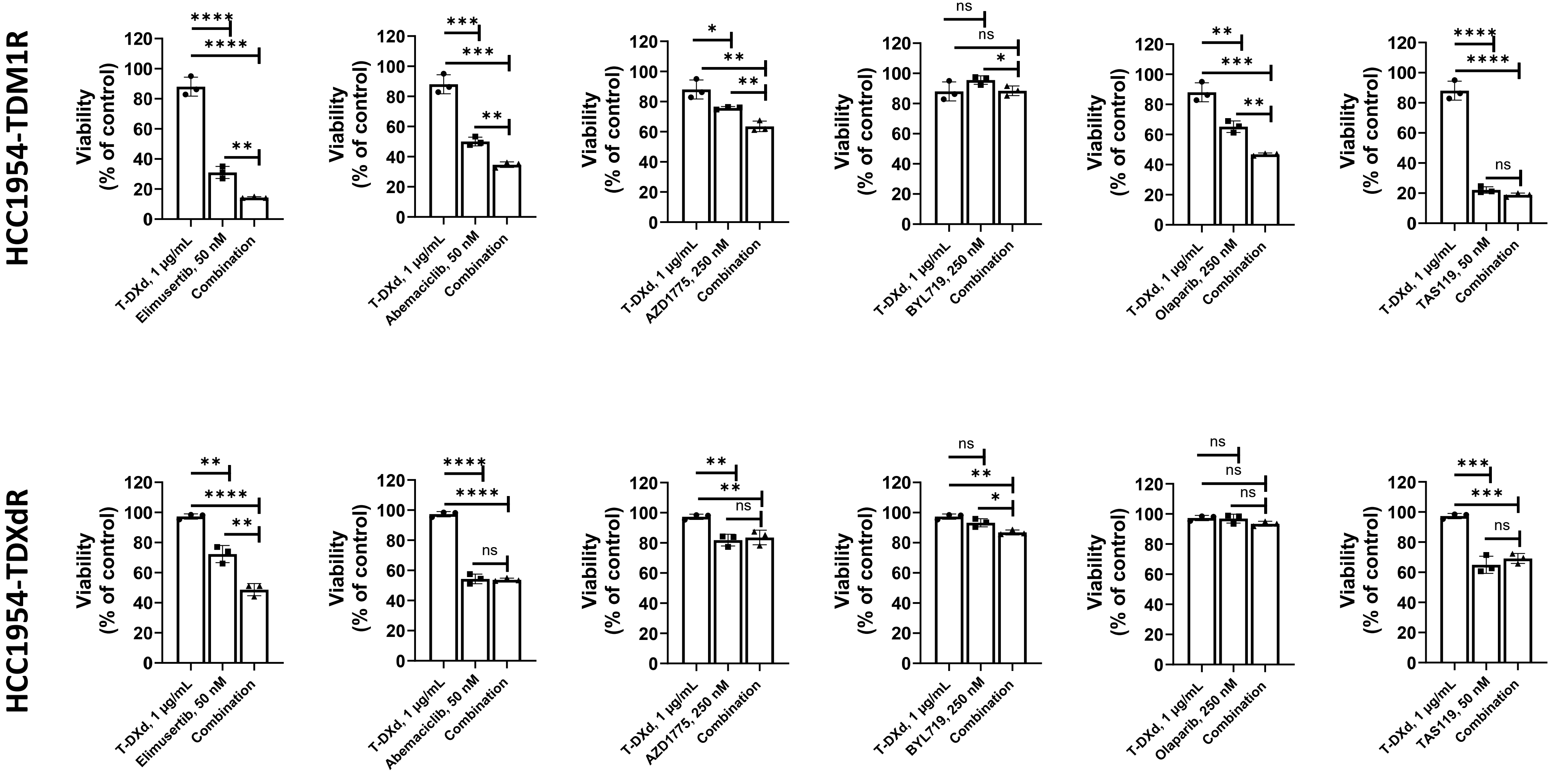

Supplemental Fig. S5

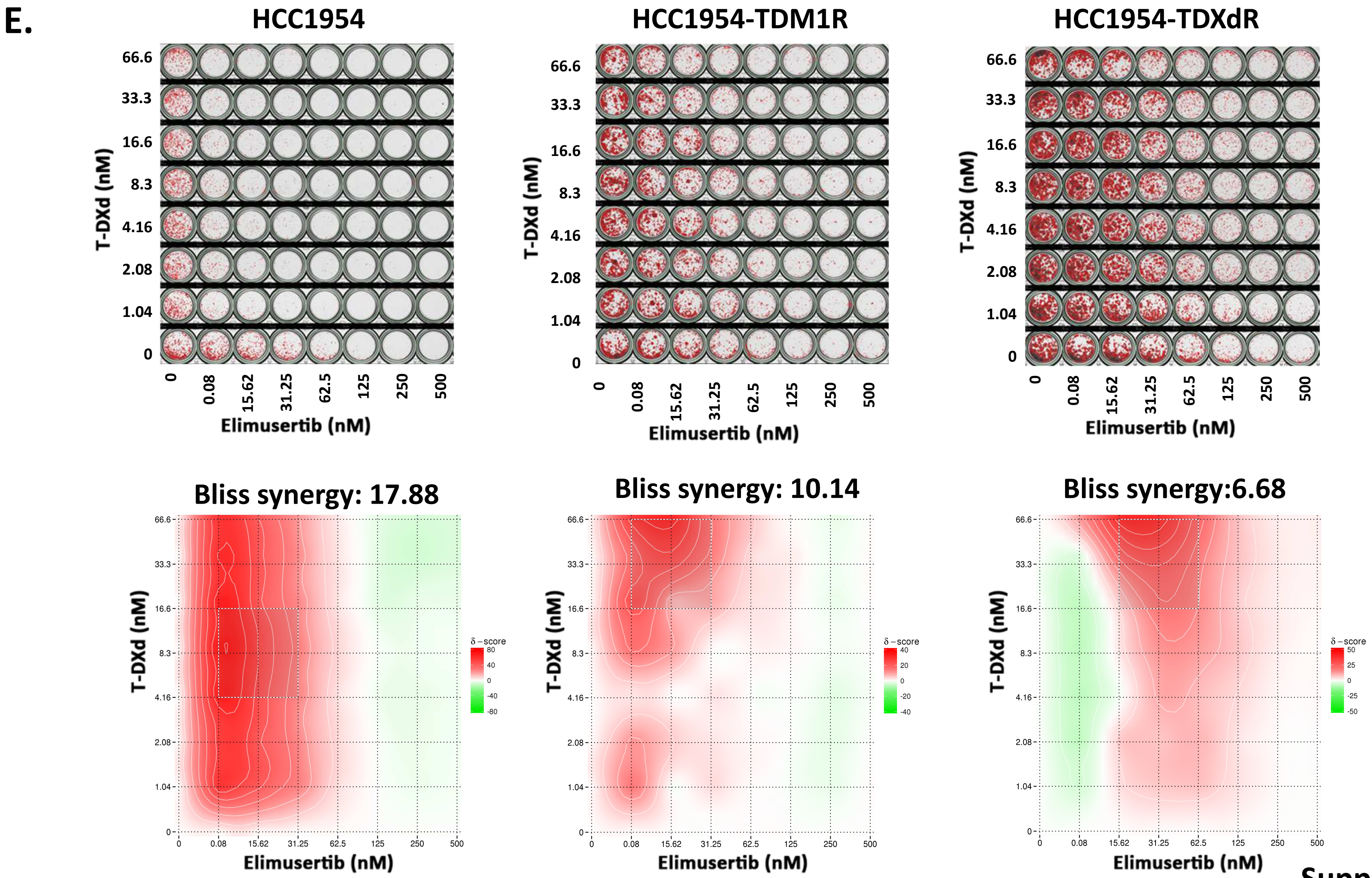

Supplemental Fig. S5

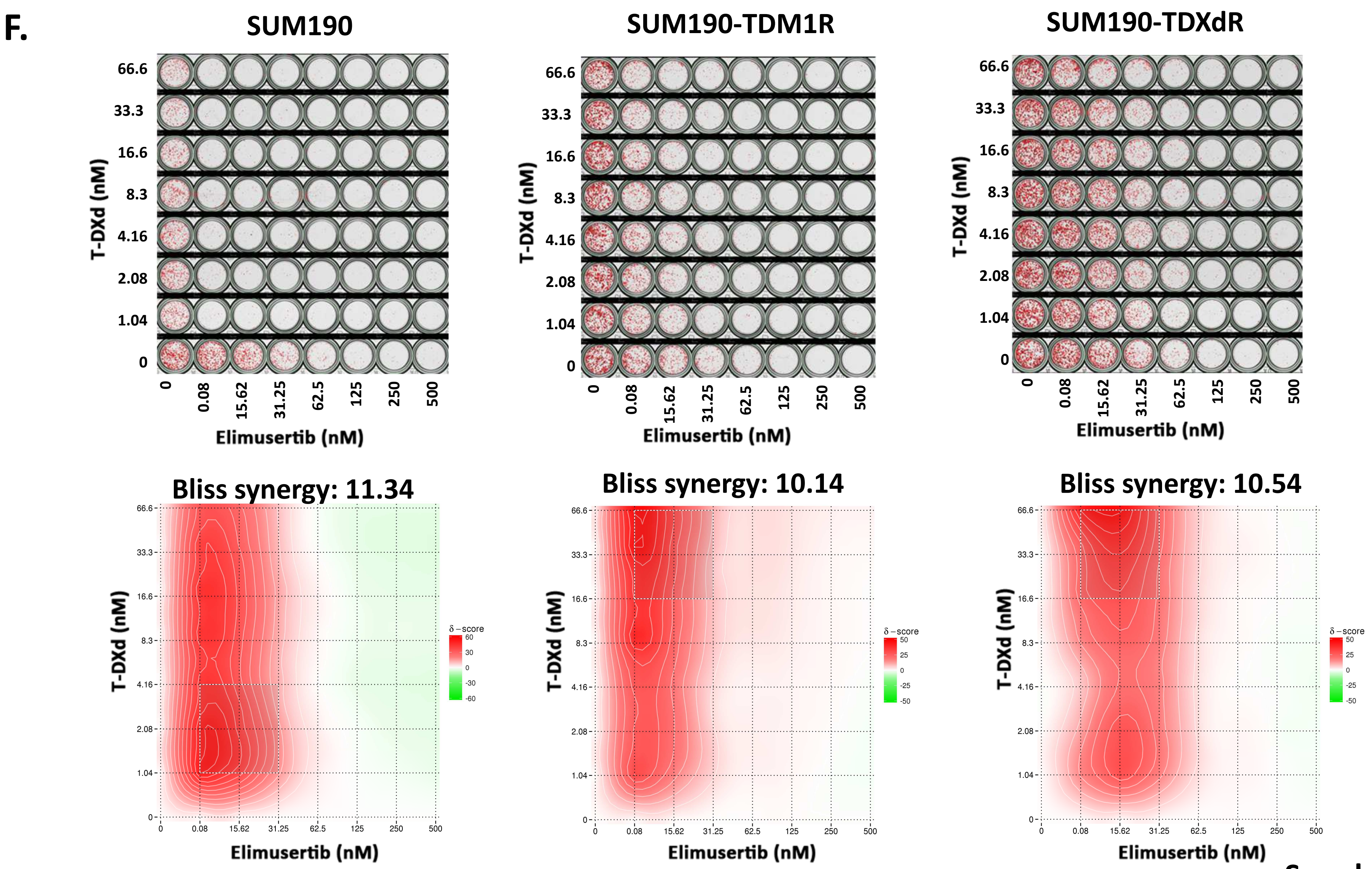

Supplemental Fig. S5

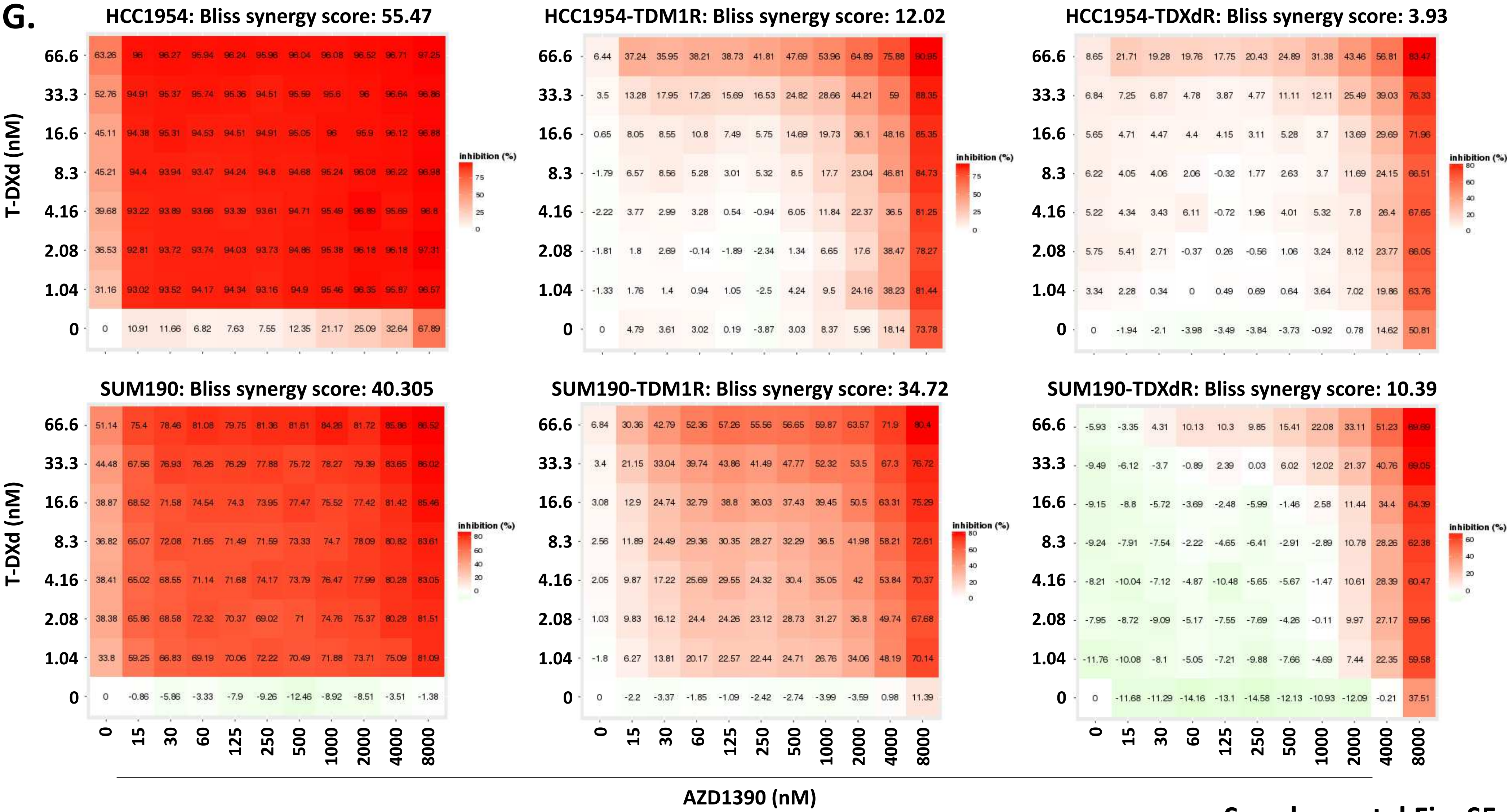

Supplemental Fig. S5

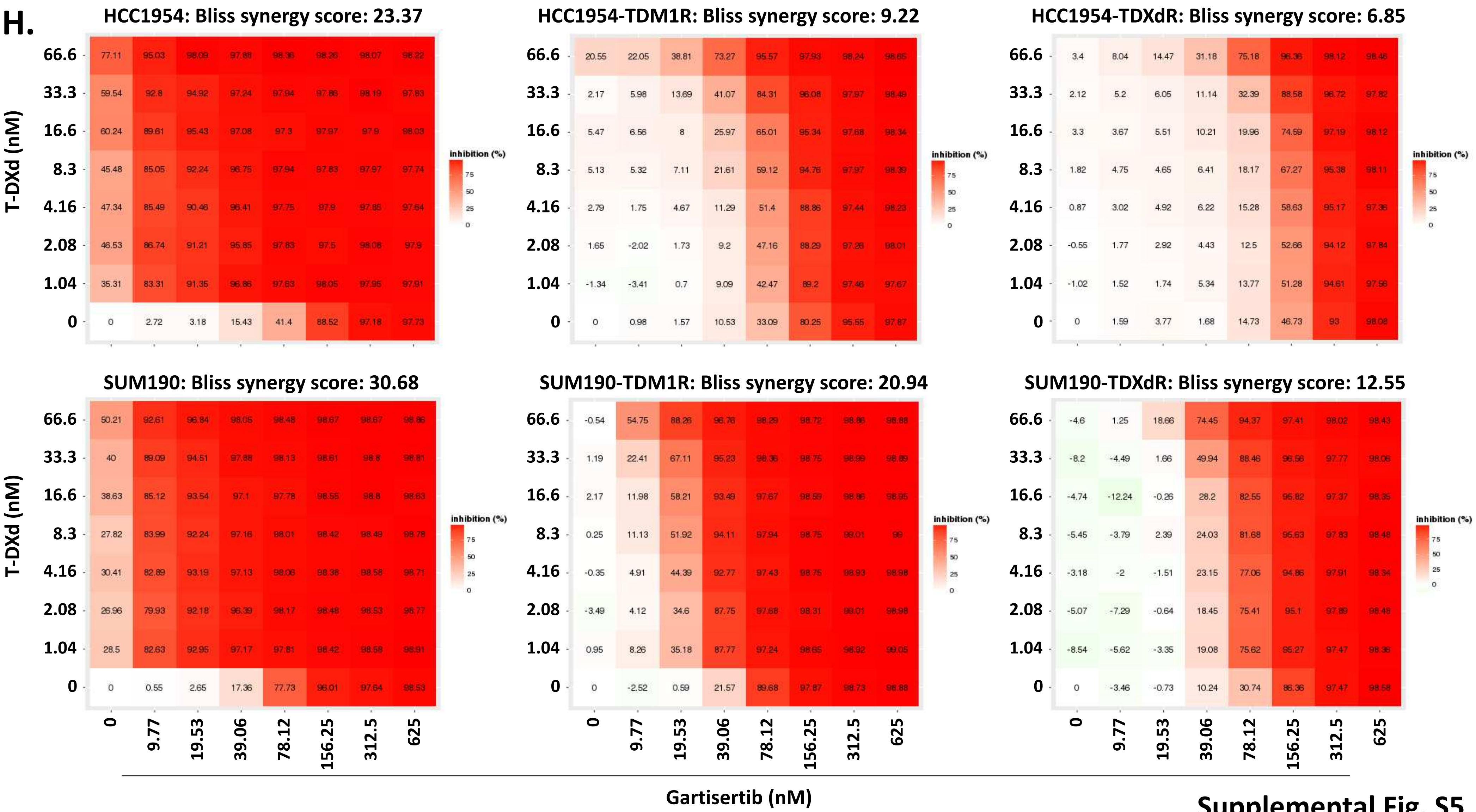

Supplemental Fig. S5

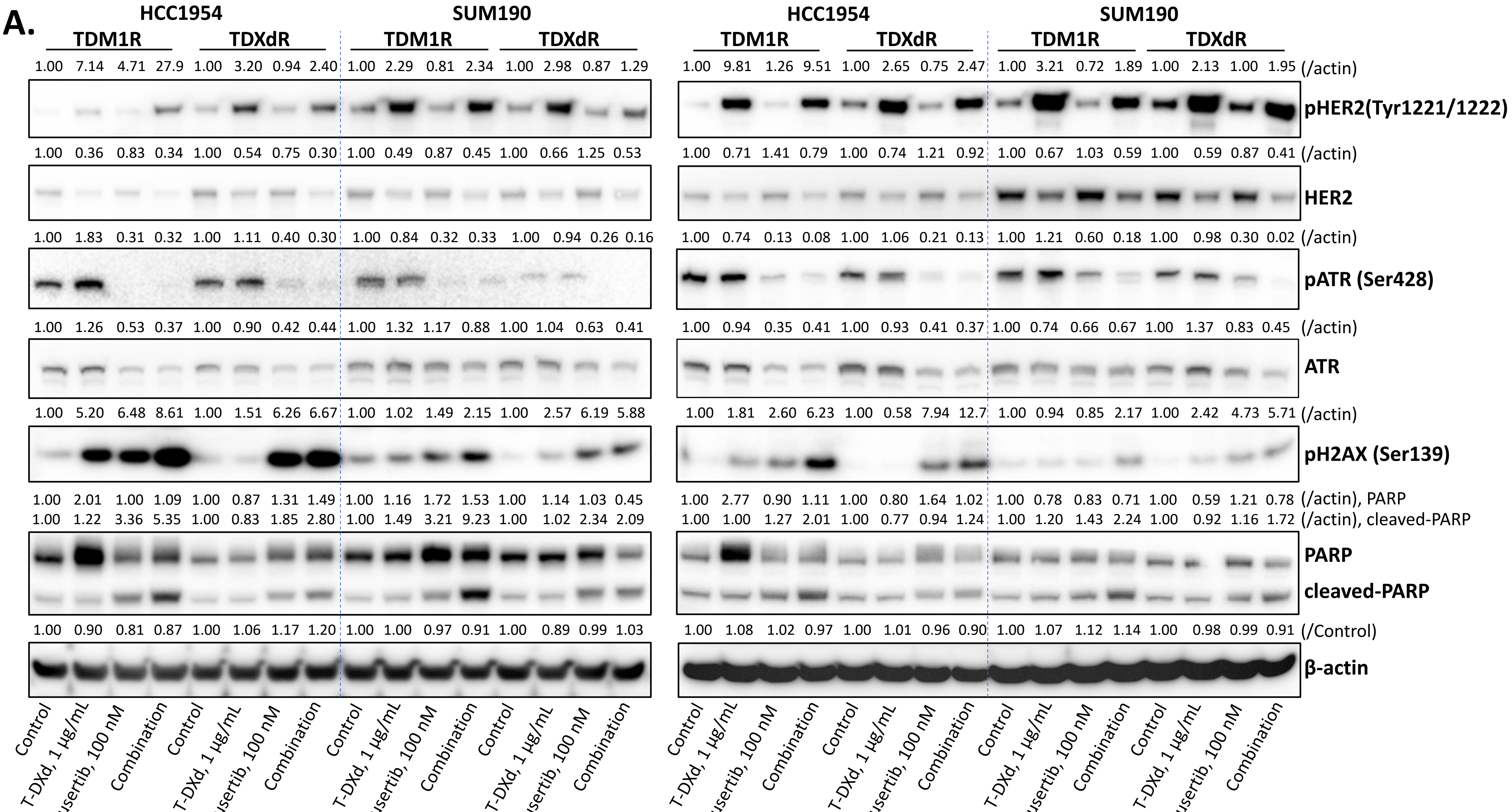

Supplemental Fig. S6

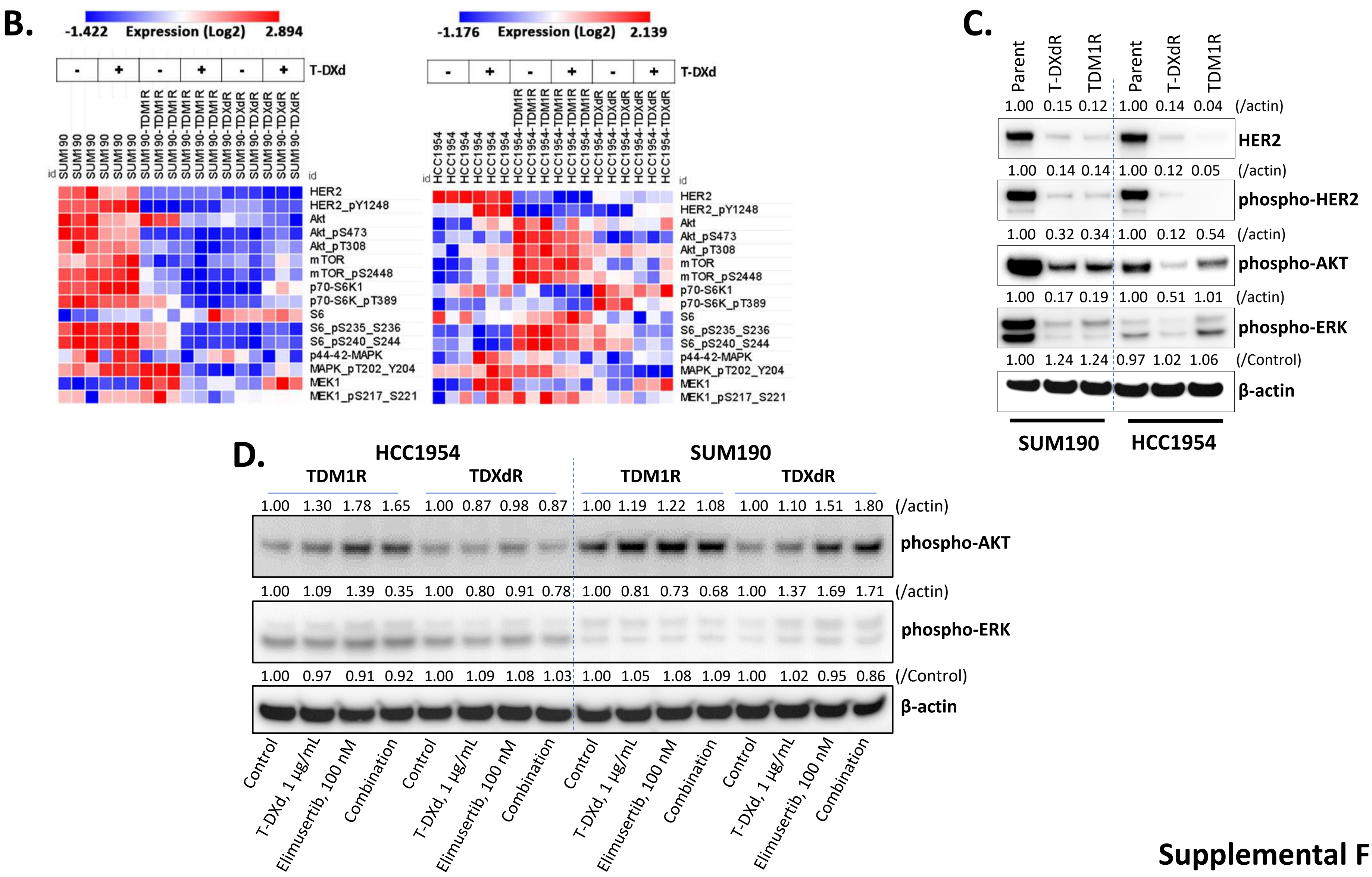

Supplemental Fig. S6

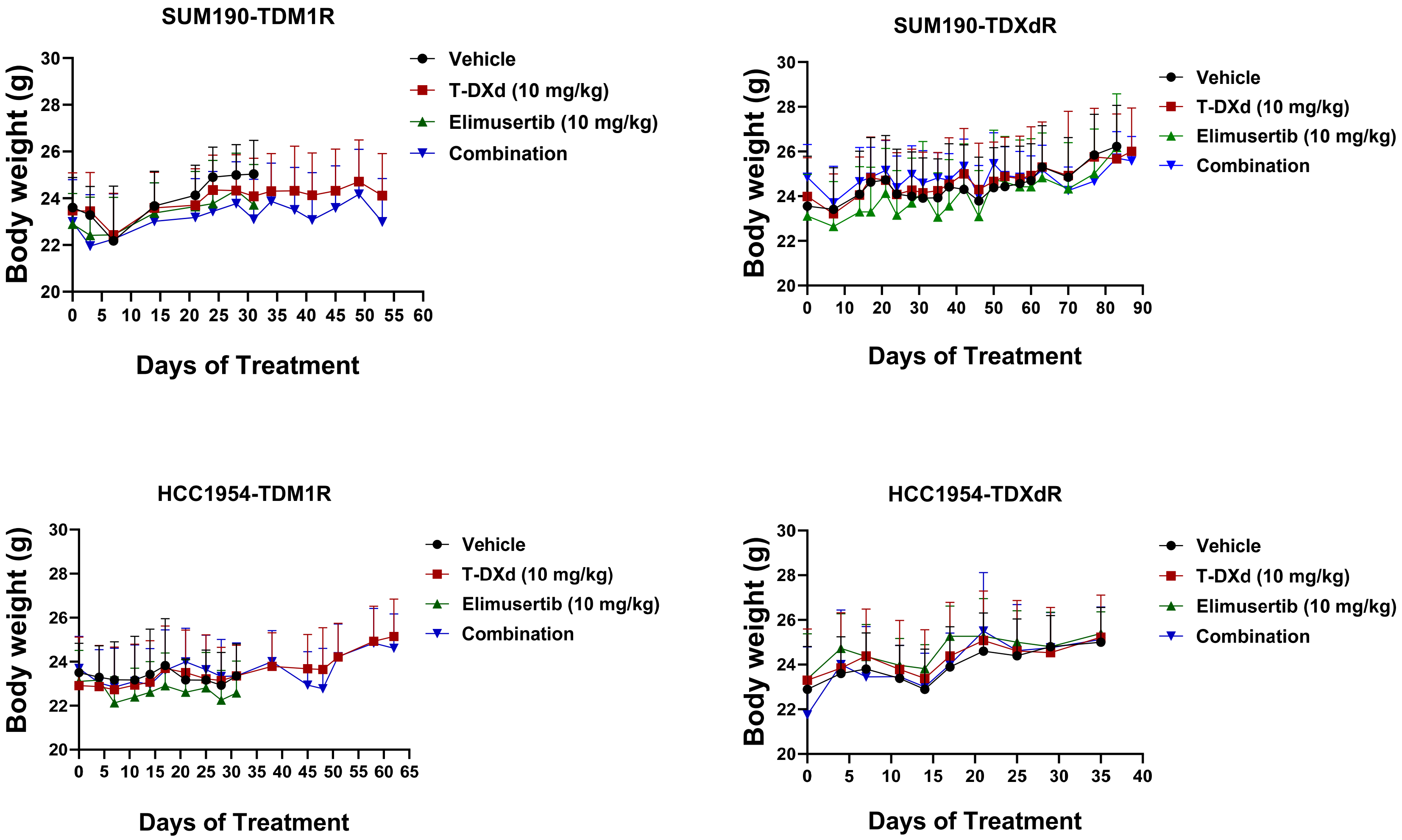

Supplemental Fig. S7

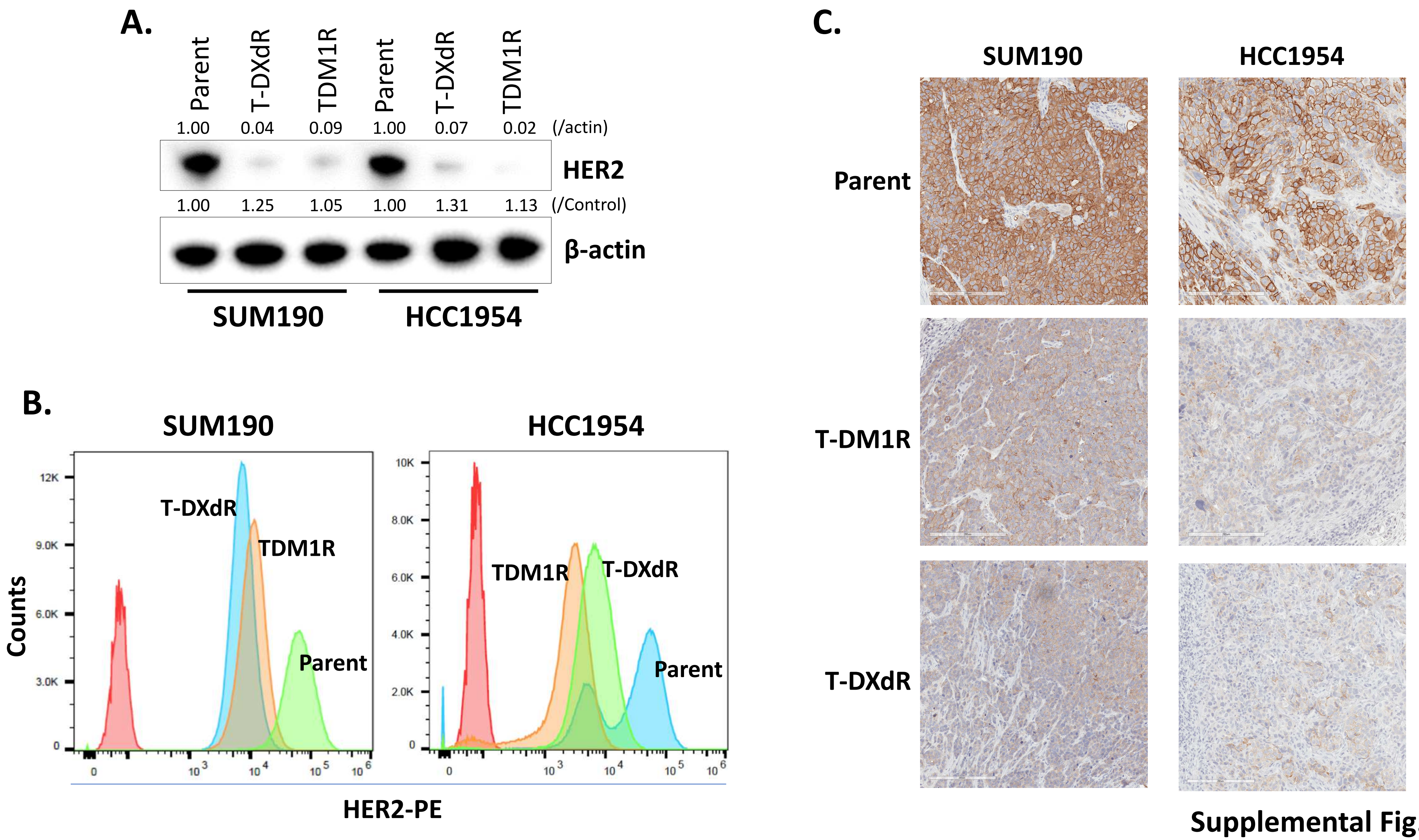

Supplemental Fig. S8

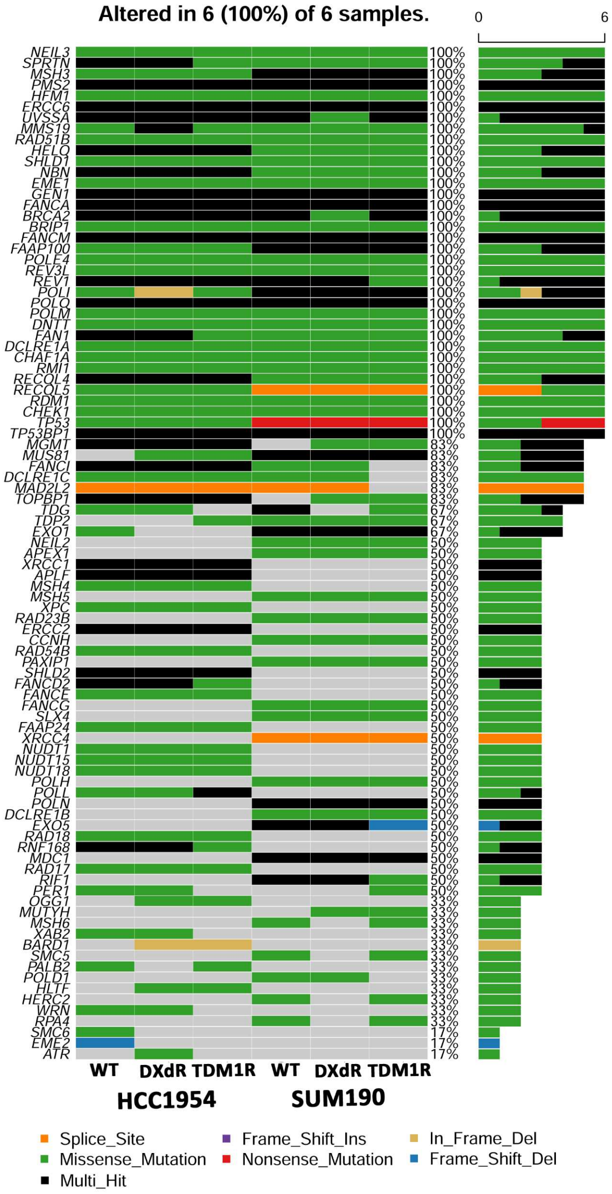

Supplemental Fig. S9
